# Glycosylation in neuroinflammation: mechanisms, implications, and therapeutic strategies for neurodegenerative diseases

**DOI:** 10.1186/s40035-025-00506-4

**Published:** 2025-09-22

**Authors:** Shenglan Cheng, Bo Xiao, Zhaohui Luo

**Affiliations:** 1 Department of Neurology, Xiangya Hospital, Central South University, Changsha, 410008 China; 2 National Clinical Research Center for Geriatric Disorders, Xiangya Hospital, Central South University, Changsha, 410008 China; 3 Clinical Research Center for Epileptic Disease of Hunan Province, Central South University, Changsha, 410008 China; 4 Research Center for Neuroimmune and Neuromuscular Disorders, Xiangya Hospital, Central South University, Changsha, 410008 China; 5 Xiangya School of Medicine, Central South University, Changsha, 410013 China; 6 Department of Neurology, Xiangya Hospital, Central South University, Jiangxi (National Regional Center for Neurological Diseases), Nanchang, 330000 China

**Keywords:** Neuroinflammation, Glycosylation, Neurodegenerative diseases, Microglia, IgG, Lectins, Targeted therapy

## Abstract

Neuroinflammation is a key pathological mechanism underlying neurodegenerative diseases, and intricately interacts with protein glycosylation. Emerging evidence suggests that aberrant glycosylation disrupts immune homeostasis, activates microglia, and promotes the release of inflammatory mediators, thereby exacerbating neuroinflammatory responses. In addition, the inflammatory microenvironment can further dysregulate glycosylation patterns, creating a vicious cycle that amplifies disease pathology. Although the regulatory role of glycosylation in neuroinflammation associated with neurodegenerative diseases has been recognized, the precise molecular and cellular mechanisms remain incompletely understood. This review systematically examines the complex crosstalk between glycosylation and neuroinflammation, with a particular focus on the critical roles of glycosylation in key neurodegenerative diseases, including Alzheimer’s disease, Parkinson’s disease, multiple sclerosis, and amyotrophic lateral sclerosis. We explore how glycosylation abnormalities contribute to disease pathogenesis through effects on immune recognition, protein aggregation, and cellular functions. Understanding the molecular underpinnings of these diseases may pave the way for the development of therapeutic strategies targeting glycosylation pathways, ultimately improving clinical outcomes for patients.

## Introduction

Neuroinflammation is characterized by aberrant activation of astrocytes and microglia accompanied by release of inflammatory mediators within the central nervous system (CNS). This pathological process has been extensively implicated in the pathogenesis of various neurodegenerative disorders, including Alzheimer’s disease (AD), Parkinson’s disease (PD), Huntington’s disease (HD), multiple sclerosis (MS), and amyotrophic lateral sclerosis (ALS) [1–5].

At the core of neurodegenerative pathology lies the abnormal accumulation of misfolded proteins. During disease progression, these altered proteins undergo structural transformation into β-sheet configurations, subsequently forming cytotoxic aggregates within neuronal cells. These proteins are exemplified by tau accumulation in AD and α-synuclein (α-Syn) aggregation in PD. Under physiological conditions, microglia maintain homeostasis by degrading these aggregates and preserving dynamic equilibrium. However, in pathological states, the excessive accumulation of protein aggregates triggers activation of microglia, promoting their transformation to the pro-inflammatory M1 phenotype. This transformation initiates a cascade of neuroimmune inflammatory responses that exacerbates neuronal degeneration and cell death [6, 7].

Glycosylation is a fundamental post-translational modification (PTM), which plays a pivotal role in the pathogenesis of neurodegenerative diseases through multiple mechanisms. It influences normal protein processing, potentially altering protein structure and function, thereby facilitating pathological accumulation. Proteoglycans expressed on immune cells can interact with glycan-binding proteins (GBPs), serving as an immune checkpoint that initiates neuroimmune responses. Furthermore, glycosylation of membrane proteins regulates autophagic processes, potentially inhibiting the degradation of protein aggregates [8–10]. Therefore, understanding the protein glycosylation alterations in neuroinflammation is important for elucidating disease mechanisms and identifying novel therapeutic targets. In this review, we comprehensively examine the role of protein glycosylation in neuroinflammation and its implications in neurodegenerative disease progression. Key molecular mechanisms are highlighted, and potential therapeutic targets are proposed to inform future treatment strategies for neurodegenerative disorders.

## Protein glycosylation regulates cellular function in the CNS

**Fig. 1:**
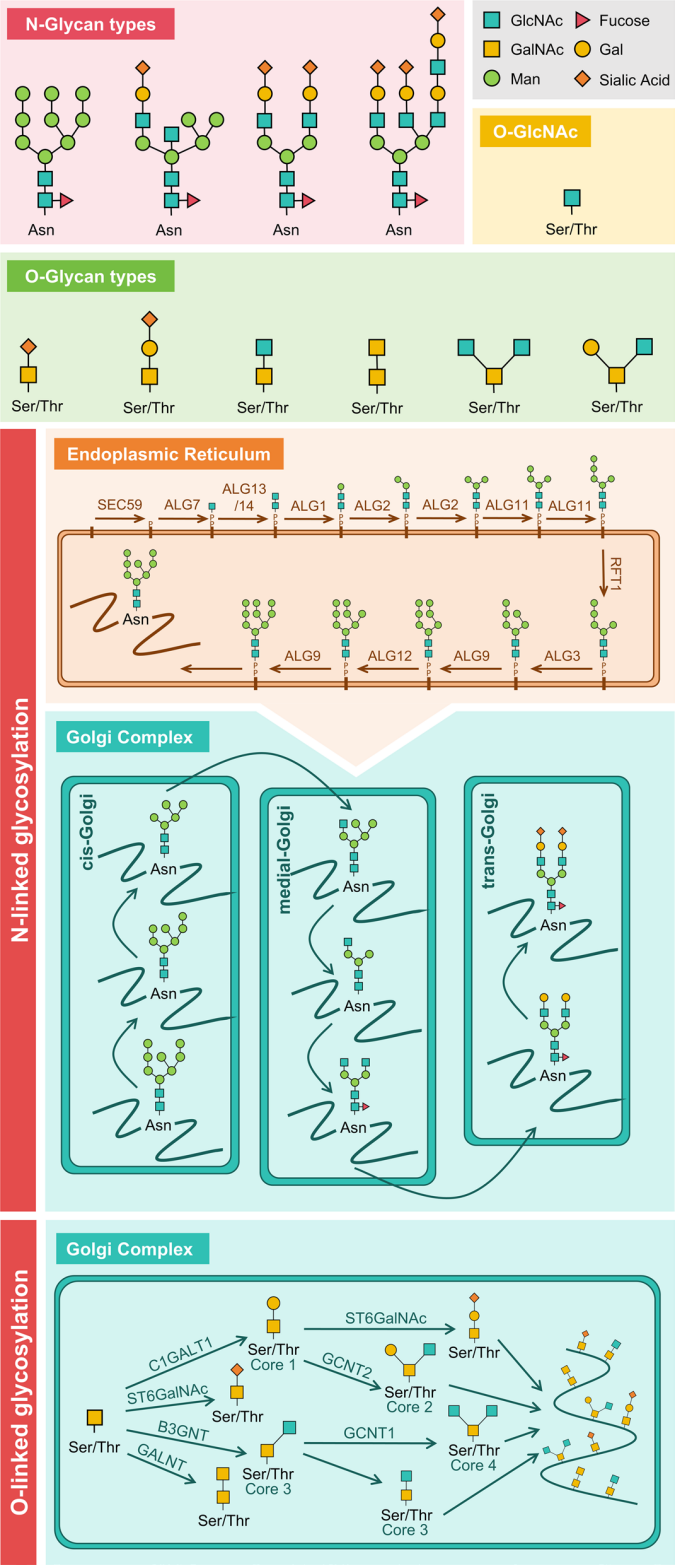
Structural features of common glycans in neurodegenerative diseases and schematic of N-linked and O-linked glycosylation processes. N-linked glycosylation begins in the ER with the assembly of a GlcNAc2Man9 oligosaccharide on dolichol pyrophosphate (Dol-PP), catalyzed by GlcNAc transferases and mannosyltransferases. Oligosaccharyltransferase transfers this precursor to asparagine residues of nascent proteins. The glycoprotein then undergoes Golgi processing, where mannosidases trim the structure to GlcNAc2Man8, followed by further modifications (e.g., galactosylation, sialylation) to form mature *N*-glycans. O-linked glycosylation occurs exclusively in the Golgi, initiating with GalNAc attachment to serine/threonine residues. Core 1 (Galβ1-3GalNAc) and Core 2 (GlcNAcβ1-6[Galβ1-3]GalNAc) structures are generated by β1,3-galactosyltransferase and GCNT1, respectively. Subsequent elongation and termination (e.g., sialylation) produce diverse *O*-glycans. Asn: Asparagine; Ser: Serine; Thr: Threonine

Glycosylation is a crucial PTM catalyzed by glycosyltransferases [11], in which monosaccharides are linked to form glycans, which subsequently conjugate with proteins or lipids, generating glycoproteins or glycolipids [12]. Depending on the linking group and the linkage, glycans in CNS can be broadly classified into *N*-glycans, *O*-glycans, and *O*-GlcNAc (Fig. 1).

Glycosylation modifications are widely present in neurons and glial cells of the CNS and play an indispensable role in maintaining normal cellular communication and physiological functions [13]. Specifically, glycosylation is critical for neural development, regeneration, synaptic plasticity, cell adhesion, signal transduction, molecular transport, cellular differentiation, and immune regulation [14, 15]. Beyond its physiological functions, glycosylation has been increasingly implicated in various pathological conditions, including neurological disorders, immune dysregulation, neuroinflammatory processes, and oncogenesis [16–19]. A prominent example is in MS, where persistent dysregulation of *N*-glycans and polysialylated neural cell adhesion molecule has been observed. This suggests a potential mechanistic link between abnormalities in glycosylation and the pathogenesis of the disease [20–22].

## Mechanisms underlying the intersection between neuroinflammation and glycosylation alteration

Emerging evidence has demonstrated a bidirectional interplay between neuroinflammation and glycosylation alterations. Activation of neuroinflammatory processes induces significant modifications of cellular and tissue glycosylation patterns, which in turn exert substantial regulatory effects on the initiation and progression of neuroinflammatory responses. The intricate molecular mechanisms underlying this reciprocal relationship warrant a comprehensive investigation to elucidate their pathophysiological implications [23].

### Abnormal glycosylation of GBPs leads to dysregulation of immune recognition

Aberrant glycosylation of GBP can alter the antigenic properties of the GBP and induce self-antigen recognition, leading to dysregulation of immune recognition. Proteins with glycosylation alterations can acquire new antigenic epitopes and thus be recognized as foreign antigens by the immune system. In addition, aberrant glycosylation of immune-associated GBPs can exacerbate neuroinflammation through specific recognization of the aberrant glycosylation structures, activating inflammatory signaling pathways, and promoting the release of pro-inflammatory cytokines.

#### Changes of glycosylation modification confer antigenic properties to self-proteins

Glycans attached to proteins and lipids direct specific biological processes, including protein folding and activity, intracellular and intercellular communication, cellular developmental processes, and particularly host–pathogen interactions and immune system communication [9, 23–25]. Alterations in glycosylation may structurally modify the self-proteins, making them possess antigenic properties, which in turn lead to a disruption of immune homeostasis, driving an inflammatory response with damage to tissue structure and function.

The pathological significance of aberrant glycosylation is particularly evident in MS, where modifications in mannose trigger inflammatory responses through activation of the mannose-binding lectins (MBLs) complement pathway, an innate immune pathway that recognizes foreign pathogens. MBL, a C-type lectin predominantly synthesized by microglia, belongs to the collagen lectin family and plays a crucial role in innate immunity. Through its carbohydrate recognition domains (CRDs), MBL recognizes specific glycan structures on pathogen surfaces and disease-modified autoantigens, including mannose and GlcNAc. This recognition triggers conformational changes in MBL-associated serine protease (MASP), such as MASP-1 and MASP-2, which initiate the complement cascade by cleaving C4 and C2 to produce C3 convertases, while simultaneously enhancing the phagocytic activity of microglia [26, 27]. Interestingly, in MS patients, researchers found elevated levels of high-mannose IgG glycoforms and correspondingly significantly higher levels of MBL in MS patients [27, 28]. Mannose is normally found on the surfaces of pathogens. Hypermannosylation of IgG triggers the MBL complement activation pathway, resulting in an inflammatory response.

Also in MS, deglycosylation of myelin oligodendrocyte glycoprotein (MOG) disrupts local immune homeostasis. Under physiological conditions, MOG is modified by fucosylated *N*-glycans that interact with C-type lectin receptors, particularly dendritic cell-specific intercellular adhesion molecule-3-grabbing non-integrin (DC-SIGN) on microglia and dendritic cells. This interaction maintains immune homeostasis through enhancing IL-10 secretion and suppression of T-cell proliferation in a DC-SIGN-, glycosylation-, and Raf1-dependent manner. However, in inflammatory environments, pro-inflammatory mediators downregulate the expression of fucosyltransferase, leading to MOG deglycosylation. This modification disrupts the MOG–DC-SIGN homeostatic axis, resulting in inflammasome activation, increased T-cell proliferation, and Th-17 differentiation, thereby contributing to MS progression [22].

#### GBPs mediate autoantigen recognition in neuroinflammation

In neuroimmunity, GBPs on immune cells act as recognition receptors, initiating pro- or anti-inflammatory cascade responses. These proteins can be broadly classified into two categories: antibodies and lectins, each playing distinct roles in neuroinflammatory processes.

##### Glycosylation of the Fc terminus of IgG antibodies influences the affinity to modulate neuroinflammation

**Fig. 2:**
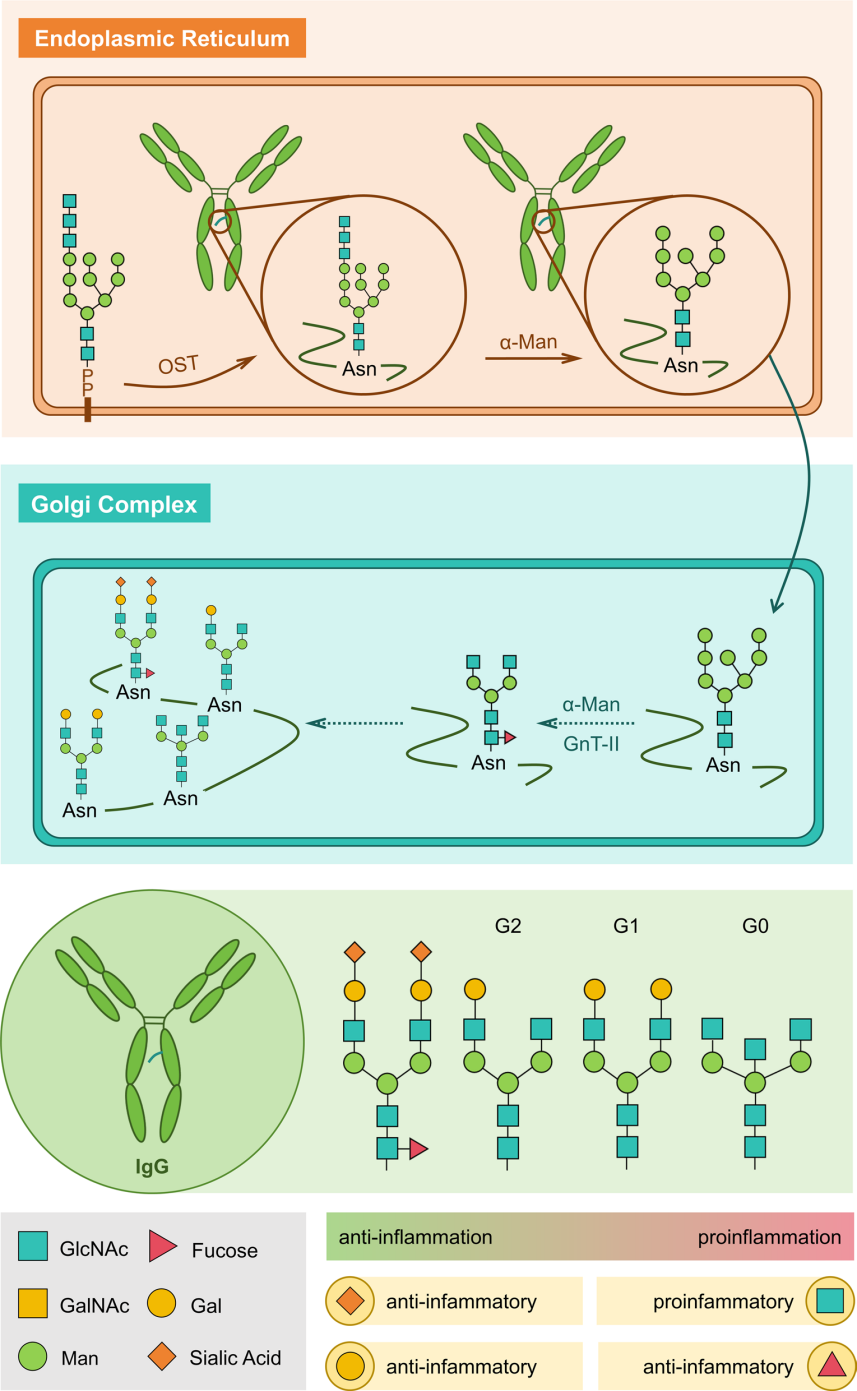
Glycosylation modifications of IgG and anti-inflammatory/pro-inflammatory properties of the corresponding glycans. IgG undergoes *N*-glycosylation in the endoplasmic reticulum, where oligosaccharyltransferase attaches Glc3Man9GlcNAc2 to asparagine residues, followed by glucose trimming to generate the Man8GlcNAc2 intermediate. This structure is subsequently processed in the Golgi apparatus through sequential actions of mannosidases and N-acetylglucosaminyltransferase I (GnT-I) to form the GlcNAc2Man3GlcNAc2 core, which is further modified by various glycosyltransferases to produce complex branched glycans. The specific glycan structures critically determine the immunomodulatory functions of IgG—core fucosylation attenuates FcγRIIIa binding and antibody-dependent cellular cytotoxicity (ADCC), terminal sialylation enhances FcγRIIb engagement to activate anti-inflammatory pathways, while bisecting GlcNAc potentiates pro-inflammatory responses through enhanced FcγRIIIa affinity. This precise glycosylation patterning represents a crucial mechanism for maintaining immune homeostasis and offers promising therapeutic opportunities for inflammatory diseases through targeted glycoengineering approaches

Antibodies are key glycoproteins in the humoral adaptive immune response and play significant roles in chronic neuroinflammation. The Fc segments of antibodies are usually modified by *N*-glycosylation, which affects their structural stability, conformation, and effector function [29, 30]. Among immunoglobulins, IgG, the most abundant immunoglobulin class in the blood, has been extensively studied for its dual pro- and anti-inflammatory functions [31]. Each IgG molecule contains a conserved *N*-glycosylation site at Asn297 in the constant weight 2 (CH2) structural domain on each of its heavy chains. This site is essential for interactions with various IgG Fc receptors and ligands, playing a central role in maintaining IgG functionality (Fig. 2) [32].

In the context of MS, glycosylation of IgG1 Fc region is altered in the inflammatory environment. For instance, in the cerebrospinal fluid (CSF) of MS patients, elevated levels of bisecting GlcNAc and reduced galactosylation are observed. These modifications alter IgG conformation, enhance its binding to Fc-gamma receptors (FcγRs), and confer pro-inflammatory properties, which are associated with intrathecal production of IgG [33]. In addition, in ischemic stroke, loss of galactose and sialic acid and increased bisecting GlcNAc, may further exacerbate IgG-mediated inflammatory responses, constituting a vicious cycle that contributes to perpetuating the inflammatory process in MS [34].

##### Lectins modulate neuroinflammatory responses through glycan-specific binding and immune regulation

**Fig. 3:**
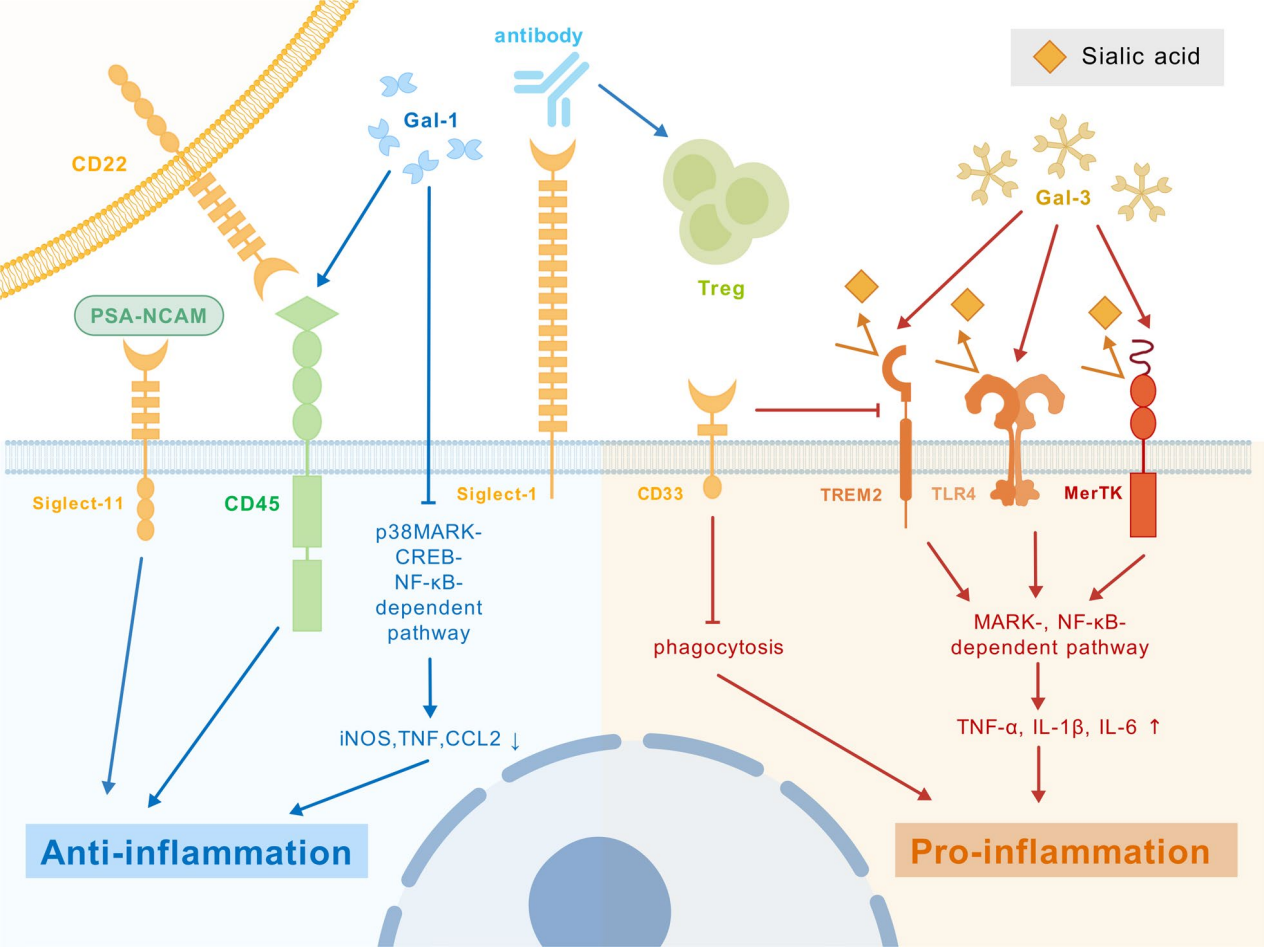
Regulatory roles of galectins and Siglecs in inflammatory responses. Galectins and Siglecs are key immunomodulators that regulate neuroinflammation through glycan recognition. Galectins bind β-galactosides: Gal-1 promotes anti-inflammatory M2 microglia by inhibiting the p38MAPK/NF-κB pathway, while Gal-3 exhibits dual roles, typically activating Toll-like receptor 4 (TLR4)-mediated inflammation but showing neuroprotection in delayed stroke treatment. Gal-9 modulates T-cell responses via TIM-3 interaction. Siglecs recognize sialic acid moieties to control immune activity. Microglial Siglec-1 mediates cell interactions, while Siglec-3 (CD33) impairs Aβ clearance in Alzheimer’s disease. Siglec-2 (CD22) contains inhibitory ITIM domains that limit B-cell activation, and Siglec-11 suppresses neuroinflammation through polysialylated ligand binding. These lectins demonstrate complex, context-dependent regulation of neuroinflammatory processes. Galectins primarily influence innate immunity through direct signaling modulation, whereas Siglecs fine-tune both innate and adaptive immune responses via inhibitory motifs. Their opposing effects—with certain members (Gal-1, Siglec-11) suppressing inflammation while others (Gal-3, Siglec-3) promoting it—highlight the delicate balance in neuroimmune homeostasis. Understanding these mechanisms offers promising avenues for developing targeted therapies against neuroinflammatory disorders, particularly through selective modulation of specific lectin-glycan interactions in disease-relevant cell types. Created with BioGDP.com

Lectins are a class of proteins that specifically bind glycans and are involved in cellular recognition, immunomodulation, signal transduction, pathogen defense, and glucose metabolism regulation. Depending on the glycans they bind, lectins can be classified as MBLs, galactose-binding lectins (galectins), GlcNAc-binding lectins, sialic acid-binding lectins (Siglecs), fucose-binding lectins, glucose-binding lectins, N-acetylgalactosamine (GalNAc)-binding lectins, sulfated glycan-binding lectins, etc. [35, 36]. In addition to MBLs that play a role in MS as previously discussed, galectins and Siglecs are key players in neuroinflammation through immune recognition (Fig. 3).

Galectins specifically recognize and bind to β-galactoside structures, including *N*-acetyl lactosamine, galactose disaccharide, and GlcNAc. Based on structural features, galectins can be classified into three groups: prototypic galectins containing a single CRD (e.g., Gal-1, Gal-2, Gal-7, etc.), tandem-repeat galectins containing two distinct CRDs connected by a linker peptide (e.g., Gal-4, Gal-8, Gal-9, etc.), and chimeric galectins containing a CRD and a non-agglutinin structural domain (N-terminal structural domain) (e.g., Gal-3). The differential CRDs allow galectins to exhibit distinct preferences for specific β-galactoside arrangements or modifications (e.g., sialylation). The expression and activity of glycosyltransferases, as well as the secretion of glycan-modifying enzymes within the Golgi apparatus, can change in response to environmental changes, including inflammation. This leads to alterations in β-galactoside composition, which in turn affects galectin sensing and function.

Among them, Gal-1 is altered in various neurodegenerative diseases, likely due to its neuroprotective functions [37]. As a key negative regulator of classically activated microglia (M1 type) activation, Gal-1 reduces the expression of downstream pro-inflammatory mediators such as iNOS, TNF, and CCL2 by targeting the activation of p38MAPK-, CREB-, and NF-κB-dependent signaling pathways. It also promotes microglial polarization toward an anti-inflammatory phenotype (M2 type), exerting neuroprotective effects. In addition, Gal-1 binds to the core 2 *O*-glycans on CD45 (a heavily glycosylated protein tyrosine phosphatase), enhancing its phosphatase activity and inhibiting the activation of pro-inflammatory signaling pathways [38]. In MS, Gal-1 has therapeutic potential by selectively targeting and promoting apoptosis of pro-inflammatory T-helper 1 (Th1) and Th17 cells [38, 39].

Gal-3 has a two-sided function. In general, it is expressed in microglia and macrophages, and promotes their activation through Toll-like receptor 4 (TLR4) binding, exhibiting pro-inflammatory properties [40, 41]. In a dextran sodium sulfate-induced acute colitis model, genetic deletion or pharmacological inhibition of Gal-3 significantly attenuated colitis severity, reduced production of pro-inflammatory cytokines (including IL-1β and tumor necrosis factor alpha [TNF-α]) and suppressed NLRP3 inflammasome activation [42]. In rheumatoid arthritis, Gal-3 is markedly overexpressed in synovial tissues, where it correlates strongly with elevated levels of pro-inflammatory cytokines (IL-6, TNF-α, and IL-17). Notably, Gal-3 promotes the unique ability of synovial fibroblasts to produce monocyte-recruiting chemokines through activation of the PI3K signaling pathway. Conversely, Gal-3 deficiency protects against antigen-induced arthritis and significantly reduces inflammatory cell infiltration [43, 44]. In PD models, Gal-3 plays a critical role in α-Syn-induced microglial activation and subsequent upregulation of pro-inflammatory mediators (iNOS, IL-1β, and IL-12) [45]. In HD models, Gal-3 is strongly associated with microglial activation and mutant Huntingtin protein aggregation, triggering inflammation through NF-κB-dependent and NLRP3 inflammasome-dependent pathways [46].

Interestingly, in stroke, delayed delivery of Gal-3 exerted anti-inflammatory properties. Kriz et al. recently revealed that Gal-3 is upregulated in proliferating microglia as early as 48 h after middle cerebral artery occlusion (MCAO), and that persistent Gal-3 deficiency specifically increases levels of the pro-inflammatory factor IL-6, exacerbating the magnitude of ischaemic injury [47, 48]. Whereas previous studies have shown crosstalk and synergy between IL-6 and insulin Like growth factor receptor 1 (IGF-R1), coimmunoprecipitation experiments suggest that Gal-3 can bind to IGF-1R [49]. Taken together, Gal-3 may exert anti-inflammatory effects by interfering with the IL-6–IGF-R1 crosstalk, which is also consistent with the neuroprotective properties exerted by a subpopulation of proliferating microglial cells co-expressing Gal-3 and IGF-1 after stroke [50]. Similarly, in the SOD1^G93A^ mouse model of ALS, Gal-3 deficiency resulted in accelerated disease progression and increased levels of TNF-α and oxidative stress, suggesting that Gal-3 plays a protective role through anti-inflammatory signaling pathways [51]. Thus, although the mechanism is not clear, the pro- or anti-inflammatory role of Gal-3 might depend on the type of injury, the stage of disease/trauma, and the time of intervention [48]. Thus, treatment strategies for neuroinflammation should be differentiated according to the stage of disease and individual differences. For example, Gal-3 should be inhibited to treat diseases with chronic onset, such as PD, and be activated to treat acute CNS disorders, such as MCAO, in order to efficiently inhibit the level of inflammation in the CNS and to achieve an optimal therapeutic outcome. In addition, multi-target combination therapies combining Gal-3 inhibitors or agonists with other anti-inflammatory or neurotrophic drugs, as well as therapeutically guided regimens that incorporate real-time Gal-3 quantification (serum, CSF, synovial fluid), imaging-based microglial activation mapping, cytokine multiplex profiling, and patient-specific genetic risk scores, may be an important direction for future research and clinical application.

Gal-8 and -9 are also involved in the regulation of neuroinflammation [52]. Gal-8 increases the number of regulatory T cells (Tregs) and production of pro-inflammatory cytokines by binding to glycosylated ligands on the surface of T cells [53]. Gal-9 interacts with TIM-3 (T cell immunoglobulin domain and mucin domain-3), a Th1-specific cell surface molecule, induces death of Th1 cells and suppresses Th1 autoimmunity, making it a potential target for preventing chronic inflammation [54].

Siglecs are a class of transmembrane proteins that play a major immunomodulatory role through recognition of salivary acid-coated glycosylated ligands on the cell surface [35]. Siglecs typically contain an N-terminal sialic acid-binding domain, immunoglobulin-like structural domains, and an intracellular tail with immunoreceptor tyrosine-based inhibitory motifs (ITIMs) or immunoreceptor tyrosine-based activating motifs (ITAMs). ITIMs recruit tyrosine phosphatases (e.g., SHP-1 and SHP-2) to inhibit downstream signaling pathways, while extracellular domains regulate immune cell activation through sialylated-ligand binding [55, 56].

Siglec-1 (sialoadhesin) lacks both ITIM and ITAM domains and is primarily expressed on microglia and macrophages, where it mediates cell–cell interactions [57, 58]. In vitro studies have shown that blocking Siglec-1 with anti-siglec-1 antibodies (SER4 and 1C2P) on macrophages leads to increased proliferation of Treg cells, thereby reducing neuroinflammation [59].

Siglec-2 (CD22) is predominantly expressed on B cells, and contains three ITIM structural domains and one ITIM-like domain. It functions primarily as an inhibitory co-receptor for the B cell receptor, inhibiting B cell response during inflammatory processes. Deletion of Siglec-2 results in B cell hyperactivation [60]. In neurons, Siglec-2 present on axonal membranes suppresses the lipopolysaccharide (LPS)-induced production of TNFα by interacting with CD45 on microglia, thereby attenuating microglia-mediated neuroinflammatory cascades [61].

Siglec-3 (CD33) is mainly expressed on myeloid cells, including microglia and macrophages. In AD, high expression of CD33 on microglia correlates with impaired clearance of amyloid-β (Aβ) plaques. CD33 recruits inhibitory proteins through its ITIMs to suppress microglial phagocytosis, leading to Aβ plaque accumulation. Additionally, CD33 may inhibit the activity of triggering receptor expressed on myeloid cells 2** (**TREM2), and the two microglial receptors have opposite effects on Aβ pathology and microglial activity. That is, CD33 may regulate the neuroimmune response by modulating the activity of downstream TREM2 [62].

Siglec-11 plays a critical role in regulating inflammation and phagocytosis [63]. When expressed on microglia, it preferentially binds to polysialylated neuronal cell adhesion molecules on neurons. This interaction leads to a decrease in the transcription of pro-inflammatory mediators, such as IL-1β and nitric oxide, in response to LPS stimulation. [64]. Thus, Siglec-11 serves as an important microglia modulator that reduces microglia-mediated neurotoxicity [65].

GBPs are crucial in regulating the function of glial cells and neurons within the CNS, exerting a significant impact on the initiation and progression of neuroinflammatory cascades. Antibodies and lectins, through their interactions with glycans, modulate immune recognition and inflammatory responses in neuroinflammatory diseases. Targeting these proteins and their associated pathways offers promising therapeutic strategies for ameliorating neuroinflammation.

### Glycosylation modulates microglial activation and astrocyte polarization

**Fig. 4:**
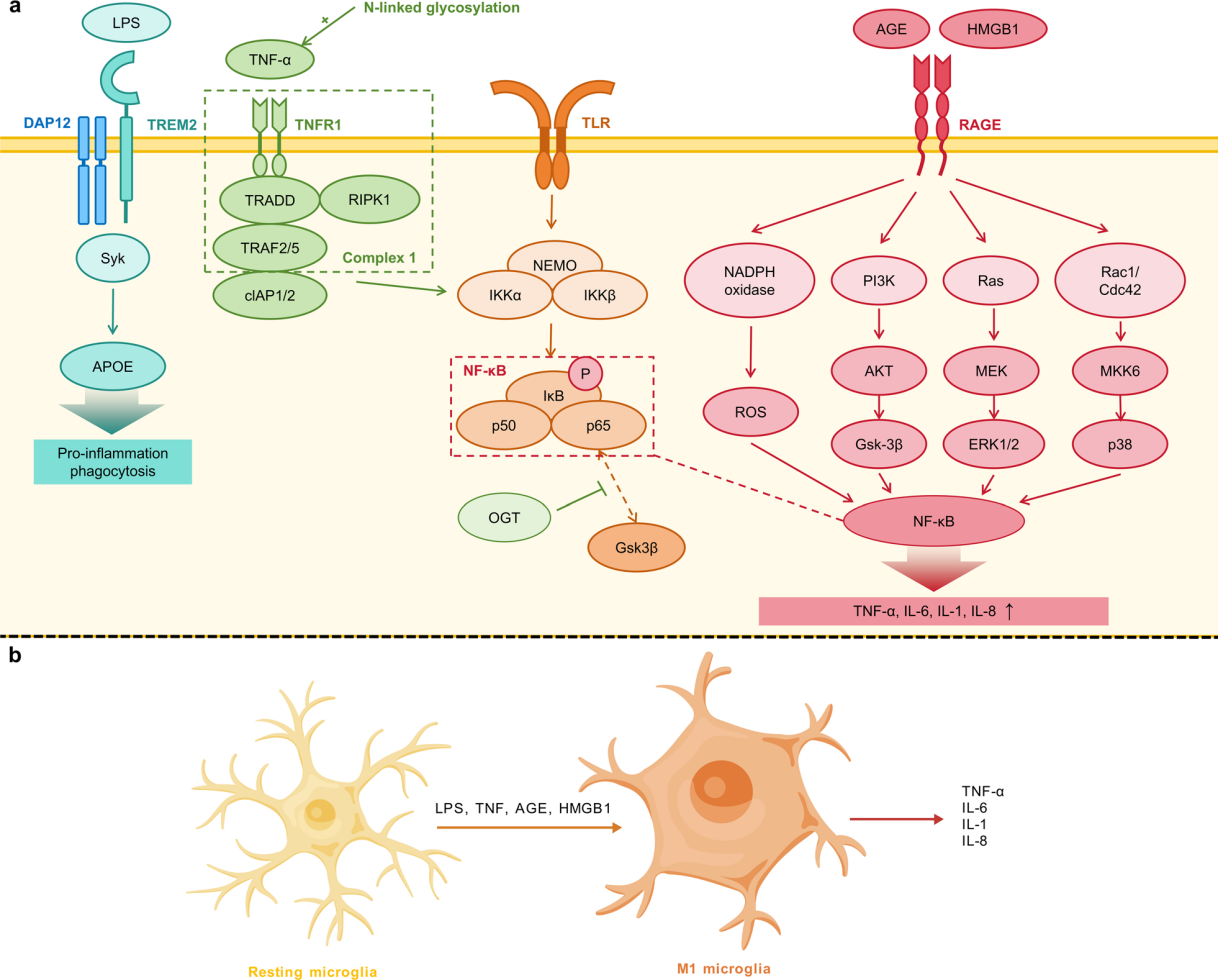
Molecular mechanisms of glycosylation-mediated modulation of microglial activation and astrocyte polarization. **a** LPS binding to TREM2 activates DAP12-Syk signaling, while ApoE4 exacerbates neuroinflammation by promoting pro-inflammatory microglial polarization through the ApoE-TREM2 axis. *N*-glycosylation of TNFR1 at Asn151/202 enhances TNF-α binding and subsequent NF-κB/MAPK pathway activation. The *O*-GlcNAcylation of NF-κB p65 by OGT inhibits Gsk3β binding, with OGT deficiency leading to increased neuroinflammation that can be ameliorated by GlcNAc supplementation. Simultaneously, AGEs-HMGB1-RAGE interactions stimulate ROS production and pro-inflammatory cytokine release via NF-κB activation. These interconnected glycosylation-dependent mechanisms collectively amplify neuroinflammatory responses in neurodegenerative diseases. The dynamic interplay between *N*- and *O*-linked glycosylation and receptor signaling pathways creates a self-perpetuating cycle of neuroinflammation, with therapeutic potential through targeting specific glycosylation events. GlcNAc administration has shown promise in reducing inflammation and improving cognitive function, highlighting glycosylation modulation as a viable strategy for treating neuroinflammatory conditions. **b** Under stimulation by factors such as LPS, TNF, AGEs, and HMGB1, resting microglia undergo the aforementioned molecular mechanisms to transform into the M1 phenotype, characterized by pro-inflammatory properties and the secretion of pro-inflammatory cytokines, including TNF-α and IL-1. Figure 4b was created with BioGDP.com

While immune recognition usually occurs on the surface of glial cells, glycosylation modifications, in addition to GBPs, act at different points of signal transduction to activate glial cells and exacerbate neuroinflammatory responses. Microglia play a central role in neuroinflammation by detecting pathological proteins, such as Aβ, and damage-associated molecular patterns (DAMPs) via pattern recognition receptors (PRRs), including Toll-like receptors, receptor for advanced glycation end product (RAGE), and NOD-like receptors. This recognition triggers downstream signaling pathways, particularly NF-κB activation, leading to the expression of inflammatory factors and initiation of inflammatory responses. Emerging evidence indicates that glycosylation modifications critically regulate multiple aspects of microglial activation and neuroinflammatory processes (Fig. 4).

On the one hand, proteins with glycosylation modification and their associated receptors are associated with neuroinflammation or will induce an inflammatory response by initiating multiple signaling pathways. RAGE is a multifunctional PRR that recognizes various ligands, including advanced glycation end products (AGEs) and HMGB1 (high mobility group box 1). Activation of RAGE triggers signaling cascades that lead to NF-κB activation, upregulation of pro-inflammatory cytokines such as TNF-α and IL-1, and production of reactive oxygen species (ROS), culminating in neuronal death. In AD, RAGE facilitates Aβ transport across the blood–brain barrier, enhances Aβ deposition, and exacerbates neuroinflammation through NF-κB pathway. Similarly, in PD, RAGE contributes to MPTP/MPP^+^-induced dopaminergic neuronal degeneration [66, 67].

On the other hand, glycosylation directly modulates signaling pathways by altering the activity of key signaling molecules. Dong et al. demonstrated that *O*-GlcNAc transferase (OGT) interacts with NF-κB p65 and catalyzes its *O*-GlcNAcylation. OGT deficiency promotes Gsk3β binding to NF-κB, leading to NF-κB pathway activation, astrocyte activation, and neuroinflammation. Notably, restoration of *O*-GlcNAcylation by GlcNAc supplementation inhibits astrocyte activation, reduces inflammation, and improves cognitive function in mouse models, suggesting a potential therapeutic strategy for neurodegenerative diseases such as AD [68].

TNF-α, a critical pro-inflammatory cytokine, mediates cell death and inflammatory responses under pathological conditions. The interaction between TNF-α and its receptor TNFR1 is essential for the activation of inflammatory cascade. *N*-glycosylation of TNFR1 at Asn151 and Asn202 notably increases its affinity for TNF-α, facilitating TNFR1 trimerization and the subsequent activation of downstream signaling pathways. This glycosylation-dependent mechanism amplifies microglial activation and neuroinflammatory responses [69].

Apolipoprotein E (ApoE), a 34 kDa glycoprotein predominantly expressed in the CNS, has three major isoforms (ApoE2, 3 and 4) with distinct *O*-glycosylation patterns. The ApoE4 isoform, which is a major genetic risk factor for AD, exhibits impaired function in modulating TNF signaling between microglia and astrocytes under LPS-induced inflammation. The increased secretion of TNF from immune cells enhances ApoE secretion levels specifically in *APOE* ε4-expressing microglia, but not in microglia from *APOE* ε2 or *APOE* ε3 mice. Conversely, TNF reduces ApoE4 secretion—without affecting ApoE2 or ApoE3 secretion—in astrocytes. These findings suggest that under inflammatory conditions, microglia-derived ApoE can suppress TNF-mediated signaling between microglia and astrocytes in an isoform-dependent manner. The impairment of this regulatory mechanism in ApoE4-expressing microglia leads to increased TNF signaling, resulting in decreased ApoE4 secretion by astrocytes [70]. Furthermore, the *APOE* ε4 allele promotes microglial polarization toward a pro-inflammatory and phagocytic state through the ApoE-TREM2 axis [71].

Intercellular adhesion molecule-1 (ICAM-1, CD54), a highly *N*-glycosylated protein expressed in CNS microglia and astrocytes, mediates leukocyte adhesion and migration through interactions with integrins (e.g., LFA-1 and Mac-1). *N*-Acetylglucosaminyltransferase III (GnT-III)-mediated modification of ICAM-1 *N*-glycans promotes NF-κB expression and stimulates the production of pro-inflammatory cytokines, including IL-1β, IL-6, and TNF-α. This process establishes a positive feedback loop that amplifies inflammatory responses [72, 73].

### Glycosylation interacts with other PTMs to regulate neuroinflammation

**Fig. 5:**
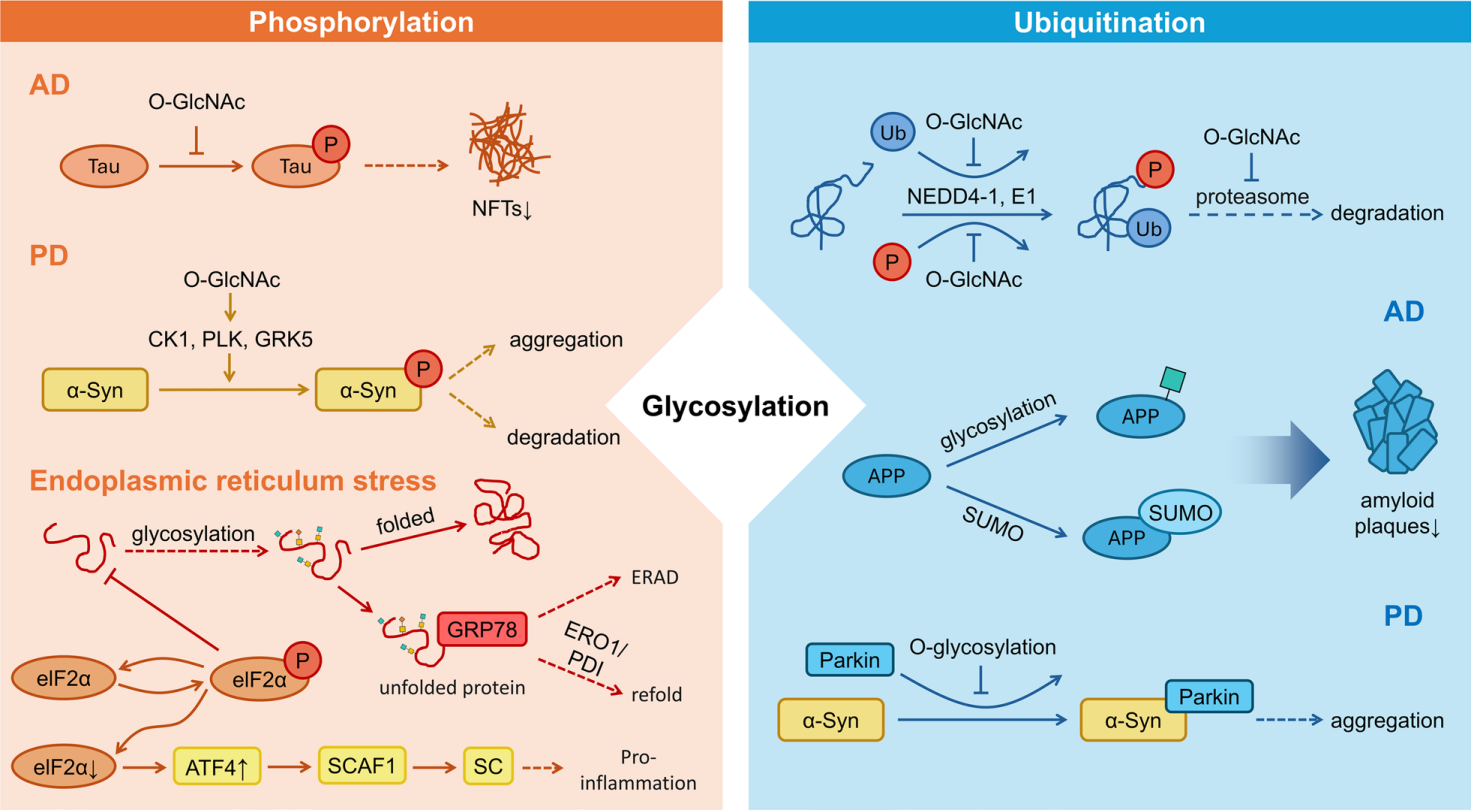
Glycosylation modifications interact with phosphorylation and ubiquitination in neurodegenerative diseases. In neurodegenerative diseases, glycosylation interacts with phosphorylation and ubiquitination to regulate neuroinflammation, protein aggregation, and stress responses. In AD, tau hyperphosphorylation promotes NFTs, while *O*-GlcNAcylation competitively inhibits phosphorylation, offering neuroprotection. In PD, kinases like CK1/PLK2 drive α-Syn aggregation via phosphorylation, whereas *O*-GlcNAcylation at Thr72 blocks it. During ER stress, glycosylation aids protein folding, while PERK phosphorylates eIF2α to suppress translation, with glycosylated ATF4 influencing cell fate. Glycosylation also modulates ubiquitination—impairing proteasomal degradation (e.g., NEDD4-1) and Parkin-mediated clearance of α-Syn in PD. In AD, APP glycosylation synergizes with SUMOylation to reduce Aβ. Thus, glycosylation is a key regulatory hub, coordinating crosstalk between PTMs in neurodegeneration

Glycosylation represents a crucial PTM mechanism that extensively interacts with other PTMs, including phosphorylation, ubiquitination, and acetylation. These intricate cross-talks critically modulate the initiation, progression, and resolution of neuroinflammation through coordinated regulation of inflammatory signaling pathways (Fig. 5).

#### Glycosylation affects the neuroinflammatory response by competing for phosphorylation sites and regulating ER stress

Phosphorylation is a fundamental PTM that involves the covalent attachment of a phosphate group (–PO₄^2^⁻) to Ser, Thr, or Tyr residues, dynamically regulating protein activity, stability, and interactions. This process is reversible under the actions of kinases and phosphatases, and is central to cellular signaling, implicated in diverse physiological and pathological processes, including metabolism, proliferation, apoptosis, inflammation, and neuroplasticity [74]. In neuroinflammation, phosphorylation frequently competes with glycosylation (e.g.,* O*-GlcNAcylation) for shared or adjacent Ser/Thr sites, thereby modulating key inflammatory pathways such as the NF-κB pathway. Targeting the phosphorylation–glycosylation interplay has emerged as a promising therapeutic strategy for neuroinflammatory disorders.

On the one hand, glycosylation directly influences protein aggregation by antagonizing phosphorylation. In AD, hyperphosphorylation of tau drives neurofibrillary tangle (NFT) formation, whereas *O*-GlcNAcylation inhibits this process by competitively occupying phosphorylation sites [75]. Similarly, in PD, site-specific *O*-GlcNAcylation at Thr72 of α-Syn not only impedes aggregation but also affects phosphorylation by kinases such as casein kinase 1, polo-like kinase, and G-protein-coupled receptor kinase 5, thereby altering α-Syn aggregation and degradation [76].

On the other hand, aberrant glycosylation also triggers ER stress, activating phosphorylation-dependent pathways that disrupt protein homeostasis and exacerbate neuroinflammation. The unfolded protein response (UPR), initiated in the ER, coordinates with the nuclear, lysosomal, and mitochondrial quality control systems to rectify misfolded proteins—a critical process for neuronal survival. During protein synthesis, nascent polypeptides undergo glycosylation followed by disulfide bond formation to achieve proper folding [77]. Misfolded glycoproteins are recognized by glucose-regulated protein 78 and directed toward chaperone-mediated refolding or ER-associated degradation via proteasomal/lysosomal pathways [78]. The calreticulin cycle and ER oxidoreductin 1 (ERO1)/protein disulfide isomerase system further mediate disulfide bond formation in misfolded glycopeptides. Notably, *N*-glycosylation is essential for the ER localization and enzymatic activity of calreticulin and ERO1 [79]. When misfolding exceeds the ER capacity, UPR-derived hydrogen peroxide disrupts the redox balance, triggering aberrant calcium release and mitochondrial apoptosis [80–83]. Concurrently, ER stress activates protein kinase R-like ER kinase (PERK), which phosphorylates eIF2α (eukaryotic initiation factor-2α) to attenuate translation, while the downstream effector activating transcription factor 4 (ATF4) upregulates SCAF1 (supercomplex assembly factor 1, also known as COX7A2L) to promote formation of respiratory supercomplexes and enhance mitochondrial respiration and stress adaptation [84]. Pharmacological inhibition of PERK in AD models (e.g., rTg4510 tau mice) restores neuronal protein synthesis and mitigates neurodegeneration [85], while ATF4 knockdown in PSEN1/APP mice preserves cholinergic neurons [86]. The ER stress further exacerbates neuroinflammation by activating JAK/STAT and NF-κB pathways in glial cells, promoting pro-inflammatory cytokine release (e.g., IL-6) and gliosis [87].

#### Glycosylation drives neurodegenerative diseases by negatively regulating protein stability through modulation of ubiquitination levels

Ubiquitination is also a critical PTM that involves the sequential actions of ubiquitin-activating (E1), ubiquitin-conjugating (E2), and ubiquitin ligase (E3) enzymes to covalently attach ubiquitin molecules to lysine residues of target proteins. Ubiquitination regulates diverse cellular functions, including proteasomal degradation, subcellular localization, enzymatic activity, and protein–protein interactions [83, 88].

Dysregulated ubiquitination contributes substantially to the pathogenesis of neurodegenerative diseases through multiple distinct mechanisms. In AD, the ubiquitin–proteasome system (UPS) removes soluble tau proteins through TRAF6 (tumor necrosis factor receptor-associated factor 6)-mediated tau polyubiquitination, resulting in degradation through the proteasome or autophagy pathway. However, abnormally aggregated tau proteins and Aβ plaques inhibit the UPS function, leading to accumulation of ubiquitinated proteins in neurons to form NFTs [89, 90]. In PD, site-specific ubiquitination of α-Syn (e.g., at Lys6/12/21) exhibits dual roles—promoting protofibril formation (enhancing toxicity) or inhibiting fibrillation—depending on the residues modified [91]. In addition, PINK1 (PTEN-induced putative phosphatase 1) activates the E3 ubiquitin ligase activity of the Ub ligase Parkin by sensing mitochondrial damage, leading to ubiquitination of damaged mitochondrial outer membrane proteins and initiation of mitochondrial autophagy. Parkin also linearly ubiquitinates NEMO (IKKγ), activating NF-κB to upregulate mitochondrial protective factors (e.g., OPA1), establishing a neuroprotective mechanism independent of mitophagy [92, 93]. In ALS, muscle-specific E3 ligases (Atrogin-1/MuRF1/TRIM32) are upregulated via FoxO3/NF-κB, degrading myofibrillar components (e.g., myosin heavy chains). Under inflammatory (TNF-α) or metabolic (insulin resistance) stress, the crosstalk between ubiquitination and autophagy accelerates proteolysis, while the proteasome-derived amino acids mediate mTORC1 activation, which paradoxically increases protein synthesis and ribosome biogenesis and suppresses autophagy, creating a vicious cycle [94–97].

Previous studies have demonstrated complex interactions between glycosylation and ubiquitination that regulate protein homeostasis through multiple-level mechanisms and play important roles in a variety of diseases. On the one hand,* O*-GlcNAc inhibits the ATPase activity of the 26S proteasome, which in turn affects the function of the proteasome and thus the ubiquitinated degradation of proteins [98]. *O*-GlcNAc modification also counteracts phosphorylation, thereby protecting the protein from ubiquitination degradation [99, 100]. *O*-GlcNAc can also regulate the activities of ubiquitination-related enzymes, such as E1 and ubiquitin ligase NEDD4-1, which in turn affects the ubiquitination process [101, 102]. On the other hand, ubiquitination may affect the function of glycosylation-associated enzymes or the glycosylation status of their substrates, which in turn indirectly affects the level and distribution of glycosylation modifications [103]. In AD, amyloid precursor protein (APP) has both *O*-GlcNAc and ubiquitination-like (SUMO) modifications. Increases in both modifications decrease the level of Aβ peptide clustering, whereas a decrease in *O*-GlcNAc modification leads to an imbalance of tau phosphorylation/ubiquitination and accelerates NFT formation [104]. In PD with Parkin mutations, unlike the physiological state in which *O*-GlcNAc-modified α-Syn can bind to the Parkin E3 ubiquitin ligase, *O*-GlcNAc-α-Syn accumulates abnormally. This suggests that Parkin and *O*-GlcNAc modification may functionally interact through the ubiquitin–proteasome pathway [105].

### Inflammatory states in turn regulate glycosylation patterns.

The glycosylation patterns within the CNS are dynamically regulated by inflammatory states, leading to significant shifts in glycan profiles. Apart from the well-documented alterations in IgG1 Fc glycosylation observed in the CSF of MS patients, characterized by elevated bisecting GlcNAc and reduced galactosylation, emerging evidence indicates that neuronal surface glycosylation is also profoundly affected by neuroinflammation [33].

Delaveris et al. demonstrated that under neuroinflammatory conditions, activated microglia have increased transcription of neuraminidase 3 (*NEU3*) and secrete NEU3 protein via extracellular vesicles (EVs). The released NEU3 modifies the neuronal glycocalyx by removing sialic acid residues. This desialylation alters the cell surface glycosylation landscape, disrupting the structural integrity of the neuronal glycocalyx. Such changes have far-reaching consequences, as the sialylation state is critical for maintaining neuronal excitability, synaptic plasticity, and network connectivity. The desialylation process triggered by NEU3 not only compromises neuronal function but also establishes a feedback loop that exacerbates neuroinflammation. The loss of sialic acid residues may further activate microglia, promoting the release of additional inflammatory factors. Additionally, this modification can influence complement system activation, potentially amplifying neuroinflammatory responses [106].

Taken together, glycosylation may be both a cause and a consequence of the inflammatory cascade response, and may be a therapeutic target for associated diseases.

## Protein glycosylation plays a role in the development of common neurodegenerative diseases.

**Fig. 6:**
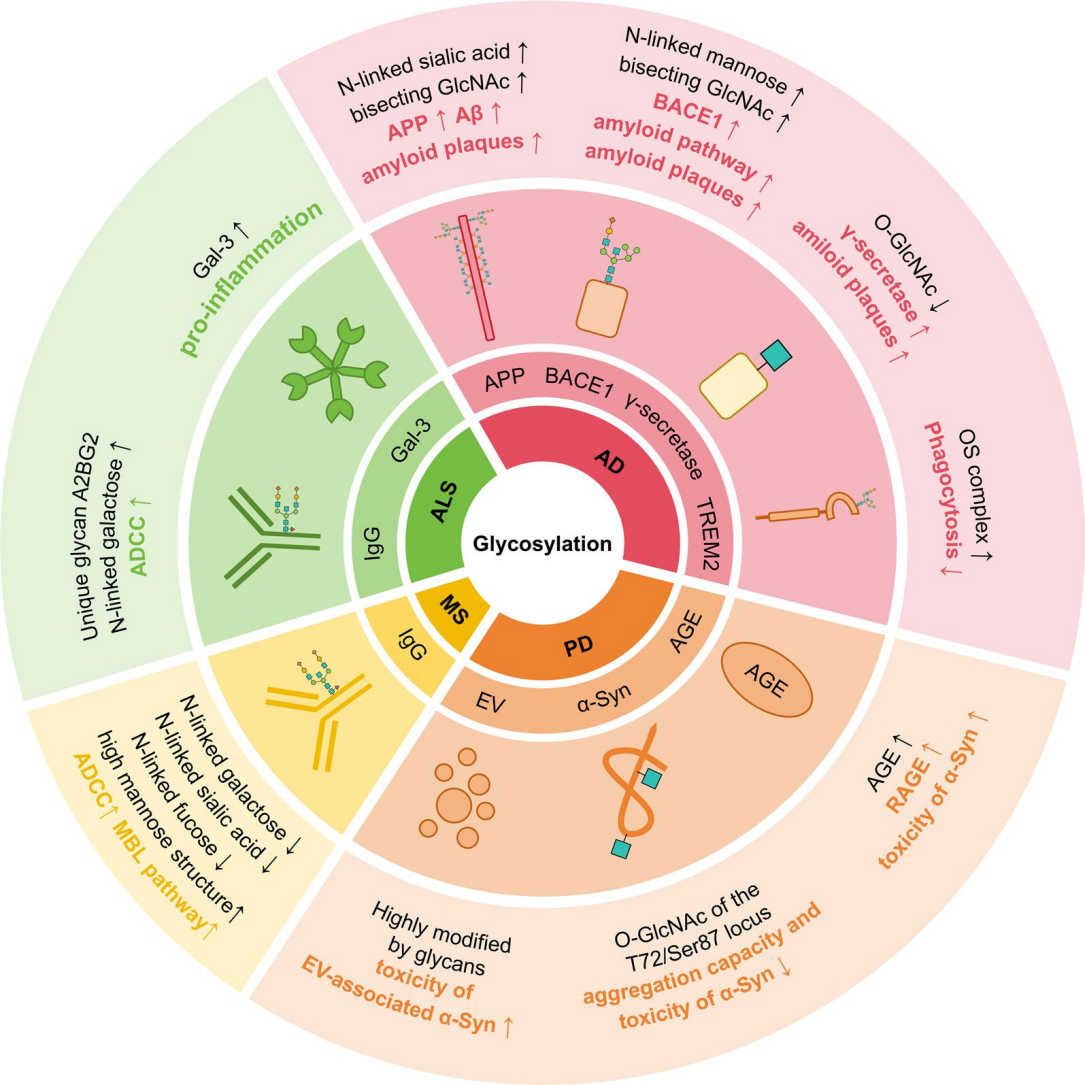
Glycosylation modifications in neurodegenerative diseases: mechanisms and implications. In AD, APP glycosylation promotes amyloidogenic processing, and the mannose-modified *N*-glycosylation enhances Aβ production by regulating BACE1 stability. TREM2 glycosylation regulates microglial activation and Aβ clearance. In PD, *O*-GlcNAcylation of α-Syn at Thr72/Ser87 inhibits aggregation, while AGEs and glycosylated EVs exacerbate its spread. MS features pro-inflammatory IgG glycosylation (reduced galactosylation/sialylation), driving demyelination. In ALS, altered IgG glycans (bisecting GlcNAc, elevated galactosylation) enhance ADCC-mediated neuron damage, and upregulation of Gal-3 promotes inflammation. These disease-specific glycosylation changes—affecting protein aggregation, immune activation, and cell-to-cell transmission—highlight glycans as central regulators of neurodegeneration. Targeting these modifications (e.g., modulating *O*-GlcNAcylation or IgG glycoforms) offers promising therapeutic strategies to disrupt the pathogenic cascades

Neuroinflammation plays a pivotal role in the onset and progression of neurodegenerative diseases, including AD, MS, and ALS [107–110]. Emerging evidence highlights that aberrant protein glycosylation contributes significantly to disease pathophysiology by modulating protein folding, immune response, and neuronal function. These glycosylation-mediated alterations not only exacerbate neuroinflammatory processes but also directly influence disease progression, establishing a complex interplay between protein glycosylation and neurodegenerative pathology [111, 112] (Fig. 6).

### AD

AD is a progressive neurodegenerative disorder characterized by memory deficits, neuropsychiatric symptoms, and a decline in the ability to perform daily activities [113]. The pathogenesis of AD involves a complex interplay of various biological processes, with its defining pathological features being the accumulation of Aβ plaques and the formation of NFTs. These pathological changes result from aberrant processing of APP and hyperphosphorylation of tau protein, respectively [113, 114]. Several studies have shown altered glycosylation patterns in AD patients, such as increased levels of bisecting GlcNAc, altered salivary acidification, decreased expression of salivary acid transferase (ST), and increased expression of the enzyme responsible for the conversion (GnT-III) [14, 115–118]. In AD, glycosylation of key AD-related proteins (e.g., APP) is altered, which further affects the glycosylation of other proteins.

#### APP glycosylation pattern influences its processing and hydrolysis

APP is a transmembrane protein widely found in neurons and is involved in cell adhesion, synaptic formation, and neuroprotection. APP undergoes proteolytic processing through two distinct pathways: the non-amyloidogenic pathway and the amyloidogenic pathway. Under physiological conditions, α-secretase cleaves APP to generate soluble APPα and C83 fragments, with subsequent γ-secretase processing yielding P3 fragments and APP intracellular domain. In contrast, the amyloidogenic pathway involves sequential cleavage by β-secretase (BACE1) and γ-secretase, producing sAPPβ, C99 fragments, and ultimately Aβ peptides (primarily Aβ40 and Aβ42). Notably, Aβ42 exhibits a higher propensity for aggregation, forming amyloid plaques that represent a hallmark pathological feature of AD [119].

APP harbors multiple *N*-glycosylation and *O*-glycosylation sites, which have been identified in the CSF of AD patients. Glycosylation plays a crucial role in regulating APP processing and trafficking. Notably, sialylation of APP *N*-glycans promotes APP secretion and Aβ production, which is associated with elevated levels of bisecting GlcNAc [120]. Also, *N*-glycosylation modulates APP transport and cellular localization, directly regulating Aβ42 levels [121, 122].

Tomita et al. found that the majority of APP cleavage by α-, β-, and γ-secretases occurs after APP undergoes *O*-glycosylation in the Golgi apparatus. This suggests that the glycosylation pattern of APP may influence its processing pathway and hydrolysis, ultimately leading to the generation of different cleavage products. Lectin-binding assays revealed distinct glycosylation patterns between α- and β-secretase-cleaved APP, indicating pathway-specific glycosylation modifications. APP contains several *O*-glycosylation sites, including mucin-type *O*-glycans and *O*-GlcNAc modifications. The latter plays a significant role in the post-translational regulation of APP [123–125]. Inhibition of *O*-GlcNAcase and an increase in *O*-GlcNAcylation may reduce Aβ production by increasing α-secretase processing, decreasing β-secretase processing, decreasing γ-secretase activity, affecting protein localization, promoting transport to the plasma membrane, and decreasing endocytosis [126–129].

#### Mannose-modified N-glycosylation promotes Aβ production by regulating BACE1 stability

Glycosylation modifications will not only change the substrate for Aβ, but will also affect the enzymes that catalyze the process. The stability and intracellular activity of BACE1, a key enzyme in Aβ production, are affected by mannose-modified *N*-glycosylation. The maturation and proper folding of BACE1 are highly dependent on *N*-glycan modifications, with the extent of *N*-glycosylation directly influencing its folding efficiency, secretion rate, and catalytic activity [86]. Liang et al., by using integrated single-cell RNA sequencing and metabolomic analyses, demonstrated that mannose enhances the stability of BACE1 and nicastrin via increased *N*-glycosylation, thereby promoting Aβ production. Notably, dietary restriction of mannose significantly reduces Aβ deposition in experimental models, suggesting a potential therapeutic strategy for neurodegenerative diseases [130].

Another study suggested that BACE1 undergoes extensive bisecting GlcNAc modification, which would stabilize BACE1 protein on oxidative stress [131]. In addition, the amount of bisecting GlcNAc on BACE1 is significantly increased in AD patients [115]. BACE1 may also affect protein sialylation by cleaving ST6GaI1 (β-galactoside α2,6-sialyltransferase-1) and down-regulating its functional transferase activity, which in turn affects APP secretion and Aβ production [120].

#### *O*-GlcNAcylation regulates aggregation and toxicity of Aβ and tau proteins

Tau, a microtubule-associated protein essential for maintaining neuronal cytoskeletal integrity and facilitating intracellular transport, undergoes significant pathological modifications in AD. Hyperphosphorylation of tau reduces its microtubule-binding capacity, leading to impaired cytoskeletal stability. Additional PTMs, including acetylation and glycosylation, further influence tau function and aggregation propensity. These aberrant modifications dramatically increase the likelihood of tau misfolding, promoting its aggregation into paired helical filaments and subsequent NFTs [132]. The deposition of NFTs disrupts neuronal cytoskeletal organization and transport systems, impairing interneuronal communication and ultimately resulting in neuronal death and brain atrophy. The synergistic interaction between pathological tau and Aβ deposition exacerbates AD neurodegeneration.

Abnormal phosphorylation of tau is widely believed to lead to the formation of NFTs. *O*-GlcNAcylation may inhibit hyperphosphorylation of tau by competitively occupying phosphorylation sites, particularly at sites such as Ser202 and Thr205, and preventing their binding to kinases. This conjecture is also consistent with the reduced levels of *O*-GlcNAcylation in AD brain extracts. Experimental data from fasting mice and a triple transgenic mouse model of AD also suggest that increased phosphorylation of tau can lead to decreased *O*-GlcNAcylation by blocking protein phosphatases [133–135]. Conversely, tau phosphorylation is also increased in neuron-specific OGT-deficient transgenic mice [136]. Therefore, abnormal tau phosphorylation may be caused by reduced *O*-GlcNAcylation and that increasing the *O*-GlcNAcylation level of tau may inhibit pathological hyperphosphorylation in AD [75, 137]. In addition, *O*-GlcNAcylation affects tau protein aggregation by directly modulating the activity of enzymes involved in tau protein phosphorylation. Glycogen Synthase kinase 3β (GSK-3β) is a key kinase involved in tau hyperphosphorylation, and its activity is regulated by *O*-GlcNAcylation, as evidenced by the fact that elevated levels of *O*-GlcNAc lead to a decrease of GSK-3β activity, which in turn decreases tau phosphorylation [75]. This is also verified by experimental results that the *O*-GlcNAcase (OGA) inhibitor Thiamet G increases the level of *O*-GlcNAcylation and correspondingly reduces tau phosphorylation [138].

Reduced levels of *O*-GlcNAcylation have also been shown to be associated with AD due to Aβ aggregation. Kim et al. demonstrated that OGA inhibitor treatment in AD mouse models significantly reduced Aβ plaque burden through decreased γ-secretase activity. This effect is attributed to the *O*-GlcNAcylation of nicastrin at Ser708, a critical component of the γ-secretase complex that functions as a substrate receptor [127].

#### Glycosylation-dependent mechanisms of glial cell activation in neuroinflammation

Microglia and astrocytes play an important role in neuroinflammation in AD. Microglia are the brain’s resident macrophages responsible for the innate immune system of CNS and play a crucial role in maintaining brain homeostasis and orchestrating protective immune responses [139]. However, in the pathologic state of AD, microglia exhibit impaired phagocytic clearance of Aβ plaques, leading to chronic activation. This persistent activation state results in the release of pro-inflammatory cytokines (e.g., IL-1β, IL-6, TNF-α), exacerbating neuroinflammation and accelerating neurodegenerative processes [139, 140].

TREM2 is expressed by microglia. It is involved in the regulation of microglia expansion, migration, survival, activation, and phagocytosis. Furthermore, it plays a key role in regulating the inflammatory response by forming a complex with DAP12 (DNAX-activating protein of 12 kDa), which recruits spleen tyrosine kinase (Syk) and activates downstream signaling pathways (e.g., PI3K-AKT-mTOR, RAS-MEK-ERK) to modulate inflammatory cytokine production and intracellular signaling [141]. The R47H missense mutation in TREM2 impairs the Syk-mediated microglial activation, reducing the Aβ plaque-encapsulation efficiency of microglia and accelerating AD pathology [142]. Furthermore, TREM2 R47H exhibits increased terminal glycosylation with complex oligosaccharides in the Golgi apparatus and decreased solubility, which affect its ligand binding and receptor function [143].

In addition, TREM2 activity can be regulated by interaction with transmembrane protein 59 (TMEM59), a type I transmembrane protein that regulates complex glycosylation, secretion, and cell-surface expression of APP, and the overexpression of TMEM59 leads to defective glycan maturation [144]. TMEM59 may play an important role in immune responses, as it has been shown to interact directly with TREM2 to regulate TREM2-dependent microglial cell activity and affect microglial phagocytic activity, migratory capacity, and metabolic status [145]. TREM2 deficiency results in impaired microglial survival and phagocytic activity, accompanied by elevated TMEM59 levels. Conversely, TREM2 overexpression promotes TMEM59 degradation. The reversal of microglial defects through TMEM59 downregulation suggests its pro-inflammatory role in AD pathogenesis, providing a potential link between glycosylation and neuroinflammation [146].

In contrast to TREM2, CD33 can promote neuroinflammation by inhibiting microglial phagocytosis [147]. As mentioned above, CD33 is expressed on microglia and its increased expression is strongly associated with increased Aβ deposition and neuroinflammation. Normally, sialylated glycans on neuronal surfaces inhibit microglial phagocytosis through interactions with CD33, protecting neurons from immune-mediated damage [148]. However, with age CD33 expression is increased in microglia, which in turn inhibits the phagocytosis of Aβ amyloid, leading to decreased clearance of Aβ plaques. When activated by Aβ, microglia release a sialidase Neu3, an enzyme that can lead to desialylation of the neuronal surface, weakening the protection of neurons against phagocytosis by microglia, which in turn leads to the over-activation of microglia and the release of pro-inflammatory cytokines and neurotoxins, exacerbating neuroinflammation [106, 149].

Gal-3, another lectin, promotes microglial pro-inflammatory polarization through its CRD by engaging galactose residues on cell surfaces and activating downstream TLR4/TREM2-mediated NF-κB and MAPK signaling pathways. Under neuroinflammatory conditions, neuronal surface sialylation is progressively lost through the action of neuraminidases (Neu1/Neu3), exposing subterminal galactose moieties that serve as high-affinity binding sites for Gal-3. This desialylation-dependent recognition mechanism enables Gal-3 to selectively target and bind to compromised neurons, further amplifying neuroinflammatory responses through its lectin-mediated interactions [150, 151].

In addition, TNF exerts isoform-specific regulation of APOE expression across glial cell types through *O*-glycosylation-dependent mechanisms. While TNF enhances ApoE4 secretion specifically in *APOE* ε4-expressing microglia (but not *APOE* ε2/ε3 variants), it paradoxically suppresses ApoE4 release from astrocytes. This divergent regulation creates a pathological feedback loop: impaired microglial-astrocytic crosstalk via the APOE-TREM2 axis drives microglial polarization toward a pro-inflammatory, phagocytic phenotype, while simultaneously reducing astrocytic APOE4 secretion[70, 71]. The resultant neuroinflammatory cascade is further amplified by *O*-glycosylation-mediated stabilization of ApoE4 in microglia, ultimately promoting neurodegenerative progression.

Microglia and their secreted exosomes also play an important role in the propagation of tau proteins. Depletion of microglia in a mouse model of induced tau protein synthesis significantly inhibited tau reproduction. Exosomes secreted by microglia play an important role in the transfer of microglial material and information exchange. In addition, higher levels of tau-containing exosomes are found in brain lysates of transgenic mice [152, 153].

#### Antibodies modified by N-linked glycan may play an important role in the clearance of Aβ and tau proteins

Emerging evidence suggests that plasma anti-Aβ IgG and peripheral inflammation are significant contributors to AD pathogenesis [154]. Glycosylation modifications play a crucial role in regulating antibody stability, immunogenicity, and functional properties, thereby influencing neuroinflammatory responses and neurodegenerative processes. Notably, structural alterations in IgG-Fc *N*-glycans can dramatically shift IgG functionality from anti-inflammatory to pro-inflammatory states. Although the precise mechanisms underlying these effects remain to be fully elucidated, strategic glycosylation engineering of anti-Aβ and anti-tau antibodies represents a promising therapeutic approach. Monoclonal antibodies against Aβ have also entered Phase III clinical trials. By enhancing antibody binding to Fcγ receptors and promoting microglial phagocytic activity, rationally designed glycosylation modifications may offer novel avenues for AD immunotherapy.

### PD

PD is primarily characterized by hallmark motor symptoms, including resting tremors, muscle rigidity, bradykinesia, and postural instability. Pathologically, PD is characterized by progressive degeneration of dopaminergic neurons in the substantia nigra pars compacta, leading to depletion of striatal dopamine. Additionally, the disease is marked by the formation of Lewy bodies, which are abnormal aggregates of α-Syn within neurons, along with persistent neuroinflammation [155]. Accumulating evidence suggests that aberrant glycosylation may contribute significantly to the pathogenesis of PD [156].

#### Glycosylation modulates α-Syn aggregation and neurotoxicity.

α-Syn is a presynaptic protein highly expressed in the CNS. It plays a critical role in synaptic vesicle transport, neurotransmitter release, and synaptic plasticity. Under pathological conditions, α-Syn undergoes misfolding and aggregation, forming oligomers, protofibrils, and fibrillar aggregates known as Lewy bodies. These processes are regulated by PTMs, including phosphorylation, ubiquitination, and glycosylation.

Methylglyoxal (MGO) is a potent glycating agent for non-enzymatic glycosylation of proteins and DNA, and promotes the formation of AGEs. Hugo et al. demonstrated that increased MGO levels induce α-Syn aggregation and enhance its neurotoxicity in vitro and in vivo, in *Drosophila* and in mice, resulting in neuronal loss and impaired synaptic transmission in mouse brain slices, and ultimately leading to motor dysfunction, reduced longevity, and decreased survival in *Drosophila*. Their study also revealed that α-Syn undergoes glycolytic modifications in the human brain. In mice, the level of α-Syn glycation increases with age, aligning with the progressive nature of PD pathogenesis [157]. These findings are supported by Morrone Parfitt et al., who identified a positive correlation between the levels of AGEs and phosphorylated α-Syn expression. The accumulation of AGEs exacerbates the burden on protein degradation and repair systems, disrupting proteostasis and contributing to disease progression [158]. Furthermore, recent studies have shown that pathological α-Syn fibers can directly bind to RAGE on microglial surfaces, activating microglia and triggering a robust inflammatory response. This interaction underscores the role of α-Syn in driving neuroinflammation in PD [159].

In contrast, structural alterations in α-Syn enable it to function as a DAMP, binding to membrane receptors on microglial surfaces. This interaction activates microglia, triggering the release of proinflammatory cytokines and ROS. Typically, this response promotes inflammation and facilitates the clearance of defective proteins. However, the phagocytic capacity of microglia is differentially regulated by α-syn forms: monomeric α-Syn enhances microglial phagocytosis, while aggregated α-Syn inhibits this process. This impaired phagocytosis contributes to the accumulation of pathological proteins and exacerbates neurodegeneration [160, 161].

Interestingly, several studies have shown that autoimmunity and neuroinflammation play a bidirectional role in the development of PD, both protective and pathogenic. Folke et al. demonstrated significantly reduced levels of anti-α-Syn IgM in PD patients, which negatively correlate with disease progression. This reduction in peripheral IgM natural antibodies leads to decreased formation of antigen–antibody complexes, impairing their clearance by macrophages and other phagocytic cells. Concurrently, PD is characterized by elevated anti-α-Syn IgG2 and reduced IgG4 levels. IgG2-antigen complexes activate the classical complement pathway, while IgG4 polymerization triggers the alternative complement pathway. These immune responses, coupled with the accumulation of pathological proteins, drive chronic localized inflammation and microglial activation, ultimately mediating antigen clearance through the complement system [162]. Researchers further elucidated the role of α-Syn-specific T cells in PD pathogenesis. They found that α-Syn accumulation, microglial proliferation, and antigen presentation lead to recruitment of α-Syn-specific T cells to the CNS. In PD patients, CD4^+^ and CD8^+^ T cells recognize α-Syn peptides presented by MHC-II on microglia and MHC-I on dopaminergic neurons. While CD8^+^ and CD4^+^ Th1 cells exert cytotoxic and proinflammatory effects, CD4^+^ Tregs provide anti-inflammatory protection against neurotoxic molecules and chronic inflammation [163–168]. Notably, adoptive transfer of copolymer-induced Th cells in MPTP (1-methyl-4-phenyl-1,2,5,6-tetrahydropyridine)-induced PD mice protects dopaminergic neurons, likely through suppression of microglial activation [169]. This bidirectional action may place greater demands on the specificity and targeting of PD-associated neuroinflammatory treatments.

While the bidirectional roles of autoimmunity and neuroinflammation in PD pathogenesis are observed at a broader, systemic level, a more targeted examination of α-Syn modification reveals that site-specific *O*-GlcNAcylation can profoundly suppress its aggregation and toxicity. Unlike the complex interplay of immune responses, this post-translational modification acts directly on α-Syn, offering a mechanistic avenue to mitigate its pathological effects. Nicholas et al. demonstrated that *O*-GlcNAcylation at the Thr72 site reduces α-Syn phosphorylation, aggregation, and toxicity in cultured cells without altering its membrane-binding or bending properties [76]. More recent research has focused on *O*-GlcNAcylation at the Ser87 site, which exhibits even more pronounced protective effects. Ser87-modified α-Syn protofibrils show a markedly reduced capacity to seed aggregation in cultured neurons and mouse brains, leading to significant decreases in pathology and toxicity. Importantly, unlike Thr72 modification, *O*-GlcNAcylation at Ser87 induces structural changes in protofibrils compared to wild-type α-Syn, suggesting a unique mechanism of action at this site [170].

#### *O*-GlcNAc may drive PD progression by regulating cellular autophagy

Autophagy is a critical cellular process for recycling or eliminating damaged proteins and organelles in response to nutrient deprivation and stress. It plays a pivotal role in maintaining cellular homeostasis [171, 172]. Dysfunctional autophagy leads to the accumulation of aberrant intracellular proteins, significantly contributing to the pathogenesis and progression of neurodegenerative diseases. The *O*-GlcNAcylation pathway, a key nutrient-sensing mechanism, dynamically responds to nutrient availability and cellular stress, thereby regulating autophagy [173].

The *O*-GlcNAc pathway is active in the brain. *O*-GlcNAc-modified proteins are abundant in nerve endings and are involved in age-dependent neuronal function [174–178]. Wani et al. demonstrated elevated levels of protein *O*-GlcNAcylation in postmortem temporal cortex lysates from PD patients. They further showed that pharmacological inhibition of OGA using Thiamet G increased *O*-GlcNAcylated proteins, suppressed mTOR and AKT activation, reduced autophagic flux, and elevated endogenous α-Syn levels [173]. Although the precise mechanism by which Thiamet G enhances mTOR and AKT phosphorylation remains unclear, one potential pathway involves the *O*-GlcNAc-mediated activation of Sp1 (specificity protein 1), which upregulates *GPAT1* (glycerol-3-phosphate acyltransferase-1) transcripts, potentially leading to mTOR activation [179].

#### Highly glycosylated exosomes mediate α-Syn transmission between neurons

EVs are ubiquitously present in biological fluids and serve as carriers for diverse biomolecules, including proteins, RNAs, miRNAs, and DNAs, playing a crucial role in intercellular communication and information transfer. Within the brain, multiple cell types, including neurons, microglia, and astrocytes, actively release EVs. These vesicles are implicated in the pathogenesis of various neurodegenerative disorders, including PD. The surface of EVs is extensively modified by glycans, which regulate their biogenesis and extracellular interactions. Notably, α-Syn-overexpressing neurons release exosomes capable of transferring α-Syn proteins to recipient neurons. This transfer facilitates the formation of α-Syn aggregates and induces cell death in recipient cells. Importantly, exosome-associated α-Syn oligomers exhibit higher cellular uptake efficiency and greater neurotoxicity compared to their free counterparts, highlighting the critical role of EVs in α-Syn propagation and PD progression [180–183].

EVs also play a significant role in modulating inflammatory cascades. Inflammasomes, multiprotein complexes in the cytoplasm, are activated by infectious or non-infectious stimuli and mediate caspase-1 activation, leading to the unconventional secretion of IL-1 and IL-18, as well as the initiation of inflammatory responses. Recent studies have revealed a bidirectional crosstalk between EVs and inflammasomes. Inflammasome activation regulates the release of EVs, which carry diverse cargos and exhibit distinct glycosylation patterns. Conversely, EVs can act upstream of inflammasome activation, either attenuating or amplifying inflammatory responses depending on the cell type producing the EVs and the specific stimuli triggering their release. This dual regulatory role highlights modulation of EV production and release as a novel therapeutic strategy to control neuroinflammatory responses [184].

### MS

MS is a chronic inflammatory demyelinating disorder of CNS, primarily affecting the brain, spinal cord, and optic nerves. MS is characterized by an autoimmune-mediated attack on myelin, leading to demyelination, axonal damage, plaque formation, and impaired nerve signal transmission. These pathological changes result in a spectrum of neurological dysfunctions, including muscle weakness, spasticity, coordination deficits, ataxia, optic neuritis, and vision loss. A key feature of MS is the presence of intrathecal IgG antibodies, whose biological activity is tightly regulated by glycosylation modifications [185].

#### Glycosylation patterns regulate IgG function to drive inflammatory response processes

Multiple studies have demonstrated reduced IgG galactosylation levels in the CSF of MS patients [33, 34]. Similar reductions in IgG galactosylation are observed in other autoimmune diseases, such as rheumatoid arthritis [186, 187].

Although IgG-Fc galactosylation was initially considered to be associated with complement activation and pro-inflammatory effects, Peschke et al. demonstrated that it does not influence antigen binding or enhance IgG affinity for FcγRIIIa. Instead, galactosylation modulates the Fc:Fc interactions among antigen-bound IgG, inhibiting hexamerization. This structural change enhances C1q binding, thereby promoting classical complement activation and complement-dependent cytotoxicity [188].

Karsten et al. demonstrated that high galactosylation of IgG N-glycans is crucial for the function of IgG1 molecules in promoting synergistic signaling between FcγRIIb and Dectin-1, activating their downstream inhibitory pathways that block pro-inflammatory effects mediated by chemokine receptors C5aR and CXCR2. This regulatory mechanism functions as a feedback loop to control complement- and chemokine-mediated inflammation in autoimmune and infectious conditions, particularly before IgG transitions to less favorable agalactosylated forms [189].

Kennedy et al. [34] demonstrated significantly lower levels of IgG sialylation in the CSF of MS patients compared to MS sera, and significantly higher levels of sialylated IgG in the sera of MS patients compared to sera of other inflammatory CNS disorders. Sialylation decreases IgG affinity for FcγRs while enhancing its binding to DC-SIGN and CD23. This structural modification upregulates the expression of inhibitory FcγRIIb on macrophages and diminishes IgG interactions with complement proteins or activating FcγRs, ultimately exerting anti-inflammatory effects [190, 191].

Core fucosylation of IgG Fc fragments plays a critical role in negatively regulating ADCC by sterically hindering the interaction between the Fc region and FcγRIIIa on effector cells. In contrast, a reduction or complete absence of core fucosylation enhances the pro-inflammatory potential of IgG, resulting in the hyperactivation of ADCC. This process has been implicated in the pathogenesis of various autoimmune diseases [192]. In MS, reduced core fucosylation of IgG is indicative of impaired ADCC regulation, potentially exacerbating disease progression through uncontrolled immune activation [193]. However, there is also conflicting evidence indicating that desialylation of IgG can reduce complement-dependent cytotoxicity as shown in the case of therapeutic anti-CD20 antibody BLX-300 [194]. Additionally, in the experimental autoimmune encephalomyelitis model, autoantibody-mediated demyelination relies on complement activation rather than Fc receptor signaling, despite elevated FcγR expression on microglia within MS lesions [28, 195]. Therefore, the net impact of reduced core fucosylation in the CSF of MS patients remains unclear, and its potential role in modulating FcγRIIIa activation cannot be excluded.

Another notable alteration in the IgG N-glycan profile of MS patients is the abundance of high-mannose structures. High-mannose glycans, typically abundant on pathogens, are recognized and bound by MBL, initiating the lectin pathway of complement activation. Under pathological conditions, MBL-mediated recognition triggers inflammatory responses. Previous studies have demonstrated that non-lactosylated IgG glycoforms can activate complement through interactions with MBL [196]. Elevated MBL levels have been consistently reported in MS patients, and recent evidence indicates complement activation in cortical gray matter lesions [27, 197]. This suggests that increased high-mannose IgG glycoforms may enhance MBL-mediated complement activation, contributing to demyelination and irreversible disease progression in MS.

#### Increased complexity of the N-glycanome plays a dual role in the inflammatory initiation chain

The most notable alteration in the N-glycan profile of plasma proteins in MS is the significant increase of highly branched structures, which include multiple galactose and sialic acid residues, reflecting an elevated complexity of the plasma N-glycanome. This increased glycosylation is particularly evident in haptoglobin, an acute-phase protein whose level increases during inflammation. The glycosylated and sialylated form of haptoglobin contains sialic acid-Lewis X motifs, which are recognized by selectins and play crucial roles in essential immune processes such as leukocyte rolling, adhesion, and migration [198, 199]. Adhesion molecules are integral to the pathogenesis of MS, as they enable autoreactive immune cells to bind to CNS endothelial cells and migrate across the BBB, a critical step in the onset of CNS inflammation [200]. The activation of endothelial and immune cells in the bloodstream leads to rapid upregulation and possible shedding of adhesion molecules into the serum and CSF, potentially further modifying the plasma N-glycanome composition [201].

In contrast, studies in both humans and mice have revealed that N-glycan branching negatively regulates T-cell activity through the T-cell receptor. This mechanism suppresses pro-inflammatory Th1 and Th17 responses while enhancing anti-inflammatory Treg activity. The process is mediated by antigen-presenting cells via TLR2/4 signaling, ultimately inhibiting inflammatory demyelination [21, 202–207]. Additionally, the N-glycosylated ligand galectin-3 negatively regulates microglial activity, reducing neurodegeneration in MS animal models [38]. Beyond its immunomodulatory effects, N-glycan branching and its ligand, galectin-3, directly inhibit neurodegeneration in mice by promoting neural stem cell differentiation into oligodendrocytes, facilitating primary myelination, supporting myelin repair, and enhancing neuronal survival, independent of inflammatory processes [208–210]. Therefore, the N-glycan group changes play diverse roles in MS, probably depending on the type of *N*-glycans.

### ALS

ALS, also known as Lou Gehrig’s disease, is a progressive neurodegenerative disorder characterized by muscle weakness, atrophy, and eventual paralysis, primarily affecting motor neurons. ALS is classified into sporadic and familial types, with sporadic cases constituting the majority and familial cases linked to genetic mutations. The pathogenesis of ALS involves multiple mechanisms, including glutamate excitotoxicity, mitochondrial dysfunction, impaired sodium/potassium ion pump activity, disrupted autophagy and axonal transport systems, and neuroinflammation [211]. Alterations in IgG glycosylation have been identified as a potential contributor to ALS pathology.

Studies have detected neuron-targeting IgG molecules with variable structural domains in the spinal motor neurons and pyramidal neurons of the motor cortex in ALS patients. The Fc region of IgG is essential for its uptake by motor axon terminals, suggesting that IgG-Fc glycans may play a role in ALS development [212–214]. This supports the hypothesis that ALS may have an autoimmune component, with changes in IgG-Fc glycosylation linked to autoimmunity [30, 215, 216]. Edri-Brami et al. identified a unique glycan structure (A2BG2) in the IgG of ALS patients, potentially resulting from differential expression of B-cell glycosyltransferases in the inflammatory environment. A2BG2 enhances the affinity of IgG for CD16 on microglia or infiltrating immune cells, thereby increasing ADCC [217]. In contrast, IgG in the CSF of ALS patients predominantly exhibits bisecting *N*-glycans, including proximal fucose and bisecting GlcNAc, along with elevated galactosylation levels [218]. Postmortem analyses have revealed significantly higher levels of Gal-3 in the spinal cord and brainstem of ALS patients compared to healthy controls [219].

Other glycosylation alterations associated with ALS include elevated serum levels of sialylated and core fucosylated *N*-glycans, as well as reduced *O*-glycosylation of CSF neurofilament-M. These changes hold promise as potential biomarkers for ALS diagnosis and monitoring.

## State-of-the-art technologies for glycosylation analysis in neurodegenerative diseases

In recent years, technological innovations with high sensitivity and resolution have provided novel tools to resolve the complex regulation of glycosylation in neurodegenerative diseases. These methods not only accurately identify glycosylation sites and glycan structures, but also reveal glycosylation heterogeneity at the single-cell level, providing critical insights into disease mechanisms and biomarker development.

### High-resolution mass spectrometry (HRMS)

HRMS is capable of accurately determining the mass of a molecule with very high mass accuracy (typically < 1 ppm) and resolution (> 50,000 FWHM), enabling the precise identification and quantification of compounds in complex biological samples. HRMS has become a core technology for resolving complex molecular modifications (e.g. glycosylation, phosphorylation) in the fields of glycoproteomics, metabolomics, and lipidomics.

The application of new mass spectrometers has brought a profound change in throughput, resolution, sensitivity, etc. SCIEX’s ZenoTOF^®^ 7600 System, launched in 2021, integrates electron-activated dissociation (EAD) technology and Zeno Trap enrichment to provide in-depth and comprehensive analyses of glycosylation modifications of protein drugs at three levels: intact protein, glycopeptide, and free oligosaccharides, with a high resolution, high sensitivity, and high duty cycle [220]. The Orbitrap Astral series of high-throughput mass spectrometer launched by Thermo Fisher Scientific in 2023 combines the excellent performance of the quadrupole and Orbitrap with the addition of the newly invented Astral asymmetric orbital nondestructive mass analyzer, which offers the best results in terms of detection throughput, depth of protein coverage, and sensitivity. Jager S et al. evaluated the performance of the Orbitrap Astral mass spectrometer in the analysis of plasma N-glycoproteomes, achieving MS/MS scanning speeds in excess of 200 Hz with high resolution and mass accuracy, significantly improving coverage and sensitivity for glycopeptide analysis [221].

Innovations in glycoproteomics analysis methods have provided more powerful tools for glycoproteomics research. Ye et al. in 2019 proposed Glyco-DIA, a data-independent acquisition (DIA)-based* O*-glycosylation strategy that enables high-throughput quantitative analysis of *O*-GalNAc-type glycoproteomics in complex biological samples. The method utilizes the SimpleCell Glycoproteomics Platform to generate homogeneous* O*-glycosylated chains and combines it with a virtually extended glycopeptide library to analyze multiple glycan structures and quantify *O*-glycopeptides from five different glycan variants in a single analysis without prior enrichment of glycopeptides[222]. Subsequently, in 2021, Yang et al. proposed GproDIA, a tool for DIA glycoproteomics analysis. It uses a two-dimensional false discovery rate algorithm and a glycoform inference algorithm, combined with a spectral library expansion strategy, for the precise identification and quantitative analysis of glycopeptides, which effectively solves the interference problem of co-fragmented glycopeptides, improves the accuracy and data completeness of glycopeptide identification, and enhances the accuracy and precision of quantitative analysis [223]. However, at the same time, it cannot be ignored that due to its peptide-centered analysis, GproDIA needs to handle large-scale spectral library resources. In these analyses, a large number of glycopeptides may not actually reach detectable levels in the sample, which increases the multiplexing burden and may compromise the sensitivity of the assay [223].

### Glycan microarray

Glycan microarray is an emerging high-throughput glycan detection technology. It constructs high-density glycan arrays by immobilizing multiple glycan chains on solid-phase carriers (e.g., slides, microarrays, etc.), so as to enable the glycan chains to specifically bind to relevant target molecules (e.g., lectins, etc.). Then the binding signals are analyzed with a signal detection system, to explore the interactions between glycan chains and target molecules and their relevant properties. However, it is inevitable that the chemical labeling step may cause changes to the protein conformation, which may lead to the deviation of its recognition specificity and affect the accuracy of the detection results. The application range of solid-phase arrays also has limitations, which will limit the prospect of glycan microarrays. Prospects of further development of glycan microarrays include realization of protein label-free detection and expanding the possibilities of carriers [224, 225].

Lectin microarrays, derived from glycan microarrays, are a lectin-based, high-throughput analytical technique for studying the structure and function of glycan chains. The technique works by immobilizing lectins with known specificity on microarrays, incubating them with fluorescently labeled samples, and detecting them by fluorescent labeling. Lectin microarrays have been widely used for biomarker discovery, especially in cancer research, such as the discovery of different glycosylated forms of serum α1-antitrypsin in lung cancer, and the discovery of plasma complement C3, histidine-rich glycoproteins, and kininogen-1 in colorectal cancer [226]. Due to the limited number of commercially available lectins, it may be difficult to find suitable lectins to recognize some uncommon glycan structures, and at the same time, the specificity of the experimental results is difficult to be guaranteed due to the possible cross-reactivity of the lectin binding to the glycan chains, and false-positive or false-negative results may occur [227].

### Single-cell glycosylation histology

Unlike glycan microarrays that focus on intermolecular interactions, single-cell glycoproteomics focuses on the composition, structure, and function of glycoproteins in individual cells. The procedures include obtaining single-cell samples by single-cell isolation techniques, capturing glycoproteins by chemical or enzymatic labeling, and mass spectrometry analysis to identify glycoproteins, analyze differential expression, and perform functional annotation and pathway analysis. Similarly, due to the limitation of detection technology, single-cell glycoproteomics faces many problems, such as low throughput, complex processing, and difficulty in identification, which limit its further application [228].

To tackle the problems of poor identification of low-abundance proteins and difficulty in balancing high throughput and high sensitivity in traditional single-cell proteomics, Ye Z’s team proposes an innovative single-cell proteomics workflow called "Chip-Tip", which utilizes the new ProteoCHIP EVO 96 chip for efficient preparation of single-cell samples. The new ProteoCHIP EVO 96 chip is utilized for efficient preparation of single-cell samples, which is combined with the Evosep One liquid chromatography system and Orbitrap Astral mass spectrometry to obtain high-quality proteomic data at the single-cell level and identify more proteins and PTMs through direct transfer and washing steps. This technological breakthrough not only enhances the sensitivity of the analysis but also realizes higher throughput and significantly improves the efficiency of data acquisition [228]. Particularly for glycosylation identification, the Chip-Tip method skips the additional enrichment step and reduces the analysis of low-abundance modifications compared to traditional PTM analysis methods. Using the Chip-Tip method, researchers directly detected multiple glycosylation-associated glycosyltransferases and glycosylation sites in single-cell samples [228].

## Therapeutic implications from neuroinflammation-related glucose metabolism disorders in neurodegenerative diseases

Several therapeutic strategies have been proposed for treating neurodegenerative diseases based on neuroinflammation-related glucose metabolism disorders (Table 1).

**Table 1:** Therapeutic approaches and targets related to glycosylation

Therapeutic approaches	Therapeutic targets	Specific effects	References
Silencing *BACE1* by siRNA	Aβ peptide production pathway	Decreases BACE1 expression and reduces Aβ synthesis	[231–234]
Mannose restriction (mannose-free diet or intracerebroventricular injection of mannose transport inhibitors)	Aβ peptide production pathway	Decreases stabilization of BACE1 and Nicastrin and reduces Aβ synthesis	[236]
*O*-GlcNAcylation recovery (Xixin decoction, etc.)	Phosphorylation of tau and alpha-synuclein	Inhibits phosphorylation of tau protein and alpha-synuclein; inhibits aggregation and neurotoxicity	[235]
Oral GlcNAc	Acts to increase *N*-glycan branching	Inhibits T- and B-cell-mediated inflammatory demyelination and triggers stem cell/progenitor cell-mediated myelin repair	[237]
Regorafenib and dihydroergocristine mesylat	Down-regulates bisectng GlcNAc level of ICAM-1	Block the NF-κB pathway and inhibit the inflammatory response	[239]
Inhibition of phosphodiesterase 10A		Reduces NLRP3 activation and inhibits inflammatory response	[240]
Human monoclonal antibodies against Aβ (e.g. aducanumab, lecanemab, donanemab, etc.)	Clearance of Aβ	Specifically binds Aβ, increasing Aβ clearance or decreasing its production	[241–243]
Intravenous immunoglobulin preparations	FcγRs	Trigger anti-inflammatory signaling pathways, regulate the activity and function of dendritic cells, Treg cells, Th17 cells and other cells, and block the inflammatory response	[244]

As APP is sequentially cleaved by BACE1 and γ-secretase, resulting in the generation of neurotoxic Aβ peptides, targeted reduction of BACE1 activity has emerged as a promising therapeutic approach for AD. However, the clinical efficacy of BACE1-related small molecule inhibitors is limited, with notable concerns on the off-target toxicity [229, 230]. Zheng et al. suggested that small interfering RNAs (siRNAs) could effectively block the expression of disease-causing genes, offering high target specificity, low effective doses, and a simplified drug development process, making them a promising avenue for future development [231]. Singer et al. demonstrated significant improvements in AD neuropathology by silencing *BACE1* using siRNA encapsulated in a lentiviral vector [232]. Despite these promising results, there is a major challenge in efficient, safe, and accurate delivery of siRNAs to the brain, especially via systemic injection. Nano-delivery systems present greater clinical potential for overcoming this barrier. Zhou et al. employed triple interactions (electrostatic, hydrogen bonding, and hydrophobic interactions) to stabilize targeted siRNAs, thus improving their biophysiological protection. Moreover, they glycosylated the nanomedicine, which achieved efficient BBB penetration through glycemia-controlled glucose transporter-1-mediated transport [233]. Alvarez-Erviti et al. engineered dendritic cells to express the exosomal membrane protein Lamp2b fused with a neuron-specific RVG peptide, facilitating the targeting of EVs to neurons. *BACE1* siRNA was subsequently loaded into these EVs by electroporation. Upon intravenous injection, RVG-guided EVs delivered siRNA specifically to neurons, microglia, and oligodendrocytes, leading to significant knockdown of *BACE1* mRNA and protein without nonspecific uptake in other tissues [234].

Targeting aberrant glycosylation has shown therapeutic potential in AD. As mentioned above, *O*-GlcNAcylation can exert neuroprotective effects by competitively inhibiting the phosphorylation sites of tau proteins and α-Syn and reducing their abnormal aggregation. Inhibition of OGA and increased *O*-GlcNAcylation (e.g., by using OGA inhibitors such as Thiamet G, NAG-T, PUGNAc, etc.) may ameliorate symptoms by increasing the level of *O*-GlcNAcylation. For instance, Xixin decoction treatment increases OGT expression levels in the hippocampus of SAD rats while decreasing OGA expression levels, reducing the accumulation of tau proteins, and significantly improving the behavior of SAD rats in a dose-dependent manner [235]. As *O*-GlcNAcylation is widely involved in a variety of cellular processes, systematic modulation of *O*-GlcNAcylation processes may lead to metabolic disorders or other consequences. The effects of *O*-GlcNAcylation restorative therapies have only been verified in animal experiments, making it difficult to ensure their safety. Therefore, efficient delivery of *O*-GlcNAc-modified enzymes (e.g., OGT and OGA) to specific brain regions or specific inhibition of *O*-GlcNAc-modified enzymes in target brain regions may become a new direction for the treatment of neurodegenerative diseases. Also, the discovery of tissue-specific proteins by the application of single-cell glycosylation and mass spectrometry may be helpful to improve the efficiency of tissue-specific delivery and reduce off-target effects. The application of single-cell glycosylation and mass spectrometry to the discovery of tissue-specific proteins may be useful for improving the efficiency of tissue-specific delivery and reducing off-target effects.

In addition, a mannose-free diet or intracerebroventricular injection of mannose transport inhibitors (e.g., 2,5-AM) significantly reduced Aβ deposition and alleviated cognitive deficits in AD mice, which may be related to the reduction of BACE1 and Nicastrin stability [236]. However, similar to *O*-GlcNAcylation restoration therapy, current studies are primarily based on animal models, and clinical trials have not yet been conducted. Further, the response to mannose restriction therapy may vary among individuals, and its long-term efficacy and safety in humans have not yet been fully validated. As mannose is a key substrate for protein glycosylation and is involved in a variety of cell signaling and immune regulation, comprehensive restriction of mannose faces many challenges, and the development of drugs that specifically inhibit the mannose modification of BACE1 should be a new direction for AD therapeutic research.

Oral GlcNAc has demonstrated efficacy in mitigating neuroinflammation and promoting neurological recovery in patients with MS. GlcNAc acts as a triple modulator, influencing inflammation, myelin formation, and neurodegeneration. In murine models, GlcNAc effectively crosses BBB, enhancing N-glycan branching, suppressing inflammatory demyelination mediated by T- and B-cells, and stimulating stem/progenitor cell-driven myelin repair. In a dose-escalation, open-label trial by Sy M et al., which involved MS patients with a non-relapsing course who were already undergoing treatment with glatiramer acetate, oral GlcNAc was found to significantly reduce serum levels of IFNγ, IL-17, IL-6, and IL-10. These changes were associated with improvements in neurological function in the participants [237].

The enzyme GnT-III, which synthesizes bisecting GlcNAc, is upregulated in AD patients [238]. GnT-III has been implicated in neuroinflammation, and its downregulation could alleviate neuroinflammation by reducing the levels of bisecting GlcNAc-modified ICAM-1, blocking the NF-κB signaling pathway, and promoting a shift in microglial activation from reactive to homeostatic states. Researchers have identified regorafenib mesylate and DHEC as potential candidates for downregulating bisecting GlcNAc levels, offering a novel approach to modulating the neuroimmune system [239].

Microglial autophagy defects are associated with neuroinflammatory activation. Modulation of autophagy could serve as a therapeutic target for neurodegenerative diseases. Defective microglial autophagy leads to elevated phosphodiesterase 10A (PDE10A) levels, and inhibition of PDE10A by MP-10 significantly reduced the Baf A1- and melanocortin-induced NLRP3 inflammasome activation. Thus, in addition to direct inhibition of NLRP3, PDE10A inhibitors may provide a novel therapeutic strategy for PD treatment [240].

Targeting different forms of Aβ, such as Aβ monomers, oligomers, protofibrils, plaques, etc., increasing the clearance of Aβ or decreasing its production by immunotherapies with human monoclonal antibodies, is a fastest growing area in AD treatment. Aducanumab, which was approved by the U.S. FDA for the treatment of early AD in June 2021, has entered phase III clinical trials, showing good potential in effectively reducing Aβ aggregation and delaying disease progression. Similar monoclonal antibodies that have entered the clinical trial stage include lecanemab, donanemab, etc. [241–243]. Glycosylation modification for antibodies may improve the potency of the antibodies by enhancing their affinity to the FcγR, which is a potential research direction for monoclonal antibody-based immunotherapy.

Also based on antibodies targeting chronic neuroinflammatory responses, intravenous immunoglobulin (IVIG) is thought to exert an immunomodulatory effect and is used to suppress autoimmune responses. IVIG can inhibit immune cell activation and inflammatory responses by interacting with FcγRs via its Fc fragment. This interaction upregulates the inhibitory receptor FcγRIIB and downregulates the expression of activating receptors. Sialylation-modified IVIG preparations bind specifically to Fc receptors, such as SIGNR1, triggering anti-inflammatory signaling pathways and demonstrating stronger anti-inflammatory activity. Additionally, the F(ab’)2 fragment of IVIG can bind and neutralize autoantibodies, cytokines, and complement components, thereby blocking inflammatory responses. IVIG also regulates the activity of dendritic cells, Treg cells, and Th17 cells, ultimately inhibiting the inflammatory response [244]. While previous studies have reported positive effects of IVIG therapy in AD patients, the efficacy remains inconsistent, with notable differences between IVIG preparations. Among them, only IVIG-C has demonstrated significant neuroprotective effects [245, 246].

## Conclusion

Neurodegenerative diseases, including AD, PD, MS, and ALS, represent a group of neurological disorders. Although differing in clinical presentations and pathophysiological mechanisms, they share common features, including abnormal protein aggregation and neuroinflammation. Based on these mechanisms, researchers have proposed various potential therapeutic strategies, such as inhibiting neuroinflammation through the regulation of glycosylation, targeting abnormally glycosylated proteins, and utilizing exosomes to modulate inflammatory responses. These strategies offer novel perspectives and directions for the treatment of neurodegenerative diseases. Future studies should investigate the specific roles of glycosylation in neuroinflammation and focus on developing innovative therapeutic approaches targeting glycosylation.

## Data Availability

Not applicable.

## Abbreviations

CNS: Central nervous system
AD: Alzheimer’s disease
PD: Parkinson’s disease
HD: Huntington’s disease
MS: Multiple sclerosis
ALS: Amyotrophic lateral sclerosis
PTM: Post-translational modification
GBP: Glycan-binding protein
ER: Endoplasmic reticulum
GalNAc: *N*-acetylgalactosamine
MBL: Mannose-binding lectin
CRD: Carbohydrate recognition domain
MASP: MBL-associated serine protease
MOG: Myelin oligodendrocyte glycoprotein
DC-SIGN: Dendritic cell-specific intercellular adhesion molecule-3-grabbing non-integrin
GnT: *N*-acetylglucosaminyltransferase
ADCC: Antibody-dependent cell-mediated cytotoxicity
CSF: Cerebrospinal fluid
Galectin: Galactose-binding lectin
Siglec: Sialic acid-binding lectin
TLR4: Toll-like receptor 4
TIM-3: T-cell immunoglobulin and mucin-domain containing-3
α-Syn: α-Synuclein
MCAO: Middle cerebral artery occlusion
IGF: Insulin like growth factor
Treg: Regulatory T cell
ITIM: Immunoreceptor tyrosine-based inhibitory motif
ITAM: Immunoreceptor tyrosine-based activating motif
Aβ: Amyloid-β
TREM2: Triggering receptor expressed on myeloid cells 2
LPS: Lipopolysaccharide
DAMP: Damage-associated molecular pattern
PRR: Pattern recognition receptor
RAGE: Receptor for advanced glycation end product
AGE: Advanced glycation end product
OGT: *O*-GlcNAc transferase
TNF-α: Tumor necrosis factor alpha
APOE: Apolipoprotein E
ICAM-1: Intercellular adhesion molecule-1
NFT: Neurofibrillary tangle
UPR: Unfolded protein response
ERO1: ER oxidoreductin 1
ATF4: Activating transcription factor 4
E1: Ubiquitin-activating enzyme
E2: Ubiquitin-conjugating enzyme
E3: Ubiquitin ligase enzyme
UPS: Ubiquitin–proteasome system
APP: Amyloid precursor protein
NEU3: Neuraminidase 3
EV: Extracellular vesicle
BACE1: β-Secretase
GSK-3β: Glycogen synthase kinase 3β
OGA: *O*-GlcNAcase
Syk: Spleen tyrosine kinase
TMEM59: Transmembrane protein 59
MGO: Methylglyoxal
ROS: Reactive oxygen species
MPTP: 1-Methyl-4-phenyl-1,2,5,6-tetrahydropyridine
HRMS: High Resolution Mass Spectrometry
DIA: Data-independent acquisition
siRNA: Small interfering RNA
BBB: Blood–brain barrier
IVIG: Intravenous immunoglobulin
